# 4EBP1-mediated SLC7A11 protein synthesis restrains ferroptosis triggered by MEK inhibitors in advanced ovarian cancer

**DOI:** 10.1172/jci.insight.177857

**Published:** 2024-06-06

**Authors:** Jiaxin Yin, Jianfeng Chen, Jing Han Hong, Yulin Huang, Rong Xiao, Shini Liu, Peng Deng, Yichen Sun, Kelila Xin Ye Chai, Xian Zeng, Jason Yongsheng Chan, Peiyong Guan, Yali Wang, Peili Wang, Chongjie Tong, Qiang Yu, Xiaojun Xia, Choon Kiat Ong, Bin Tean Teh, Ying Xiong, Jing Tan

**Affiliations:** 1 State Key Laboratory of Oncology in South China, Guangdong Provincial Clinical Research Center for Cancer, Sun Yat-sen University Cancer Center, Guangzhou, China.; 2 Cancer and Stem Cell Biology Program, Duke-NUS Medical School, Singapore.; 3 Department of Oncology, Guangdong Provincial People’s Hospital, Guangdong Academy of Medical Sciences, Southern Medical University, Guangzhou, China.; 4 Department of Laboratory Medicine, Guangzhou First People’s Hospital, School of Medicine, South China University of Technology, Guangzhou, China.; 5 Lymphoma Genomic Translational Research Laboratory, Cellular and Molecular Research, and; 6 Division of Medical Oncology, National Cancer Centre Singapore, Singapore.; 7 Genome Institute of Singapore, A*STAR, Singapore.; 8 Laboratory of Cancer Epigenome, Division of Medical Sciences, National Cancer Centre Singapore, Singapore.; 9 Hainan Academy of Medical Science, Hainan Medical University, Haikou, China.

**Keywords:** Oncology, Therapeutics, Cancer, Drug therapy, Translation

## Abstract

Loss of ferroptosis contributes to the development of human cancer, and restoration of ferroptosis has been demonstrated as a potential therapeutic strategy in cancer treatment. However, the mechanisms of how ferroptosis escape contributes to ovarian cancer (OV) development are not well elucidated. Here, we show that ferroptosis negative regulation signatures correlated with the tumorigenesis of OV and were associated with poor prognosis, suggesting that restoration of ferroptosis represents a potential therapeutic strategy in OV. High-throughput drug screening with a kinase inhibitor library identified MEK inhibitors as ferroptosis inducers in OV cells. We further demonstrated that MEK inhibitor–resistant OV cells were less vulnerable to trametinib-induced ferroptosis. Mechanistically, mTOR/eIF4E binding protein 1 (4EBP1) signaling promoted solute carrier family 7 member 11 (SLC7A11) protein synthesis, leading to ferroptosis inhibition in MEK inhibitor–resistant cells. Dual inhibition of MEK and mTOR/4EBP1 signaling restrained the protein synthesis of SLC7A11 via suppression of the mTOR/4EBP1 axis to reactivate ferroptosis in resistant cells. Together, these findings provide a promising therapeutic option for OV treatment through ferroptosis restoration by the combined inhibition of MEK and mTOR/4EBP1 pathways.

## Abstract 2

MEK inhibitors are potential inducers of ferroptosis, and 4EBP1-mediated SLC7A11 protein synthesis modulates MEK inhibitor–triggered ferroptosis sensitivity.

## Graphical abstract

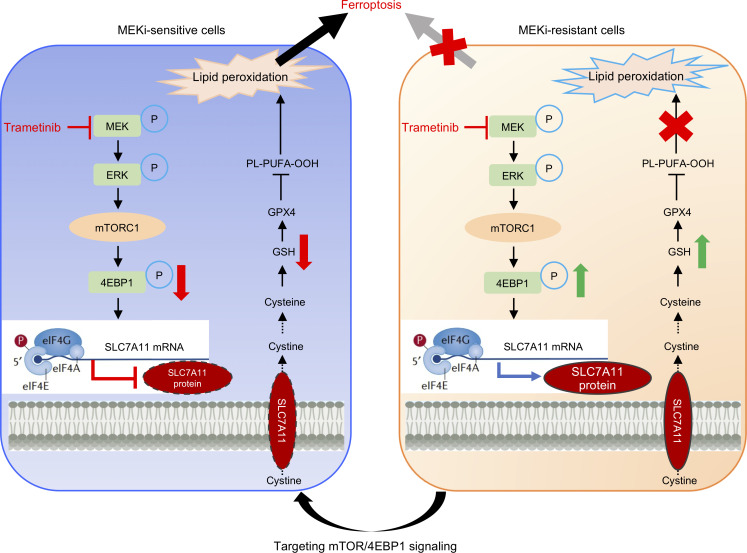

## Introduction

Ovarian cancer (OV) has the highest mortality rate among gynecological cancers (1), in part due to its late diagnosis and drug resistance (2). Late-stage diagnoses often lead to drug-resistant forms of the disease, necessitating more effective therapy. Targeted therapies, including VEGF inhibitors and poly (ADP ribose) polymerase (PARP) inhibitors, are effective only for a limited number of patients (3). Therefore, it is urgent to explore new therapeutics to improve the clinical outcomes of patients with OV.

Ferroptosis is an iron-dependent form of regulated cell death caused by excessive lipid peroxidation (4). Among ferroptosis negative regulation (FNR) genes, GPX4 is a core repressor of ferroptosis in cancer cells. It can use reduced glutathione (GSH) as a cofactor to detoxify lipid peroxidation and protect cells against membrane lipid peroxidation (5). Another FNR gene, solute carrier family 7 member 11 (SLC7A11; also known as xCT) works as an amino acid transporter to uptake extracellular cystine, followed by reduced GSH and eventually inhibition of lipid peroxidation (6). Inhibition of GPX4 and other FNR genes that suppress lipid peroxidation would predispose tumor cells to ferroptosis (5, 7, 8). Conversely, escape of ferroptosis contributes to the development of various tumors, such as hepatocellular carcinoma, pancreatic cancer, and OV (9–11), suggesting that ferroptosis inducers may be potential therapeutic strategies for patients with OV.

Among all the ferroptosis inducers, erastin is one of the most widely used compounds to trigger ferroptosis in various cancers (8). Nevertheless, due to its limited solubility and low metabolic stability in vivo, erastin has been precluded clinically (4). Meanwhile, several kinase inhibitors have been reported to induce ferroptosis in tumors. For example, sorafenib, the first multi–tyrosine kinase inhibitor approved for patients with hepatocellular carcinoma, was identified as a ferroptosis inducer (12). FGFR4 inhibitors could also trigger ferroptosis in recalcitrant HER2-positive breast cancer and hepatocellular carcinoma (13). Therefore, kinase inhibitors might be effective ferroptosis inducers as potential therapeutic strategies for patients with OV.

Herein, we reported that loss of ferroptosis played a crucial role in the tumorigenesis of OV and identified MEK inhibitors as potential inducers of ferroptosis. We unraveled a potential mechanism for the escape of ferroptosis triggered by MEK inhibitors, which is by promoting SLC7A11 protein synthesis. We found that targeting mTOR/eIF4E binding protein 1 (4EBP1) signaling could restore ferroptosis through inhibition of SLC7A11 protein synthesis. Therefore, cotargeting MAPK and mTOR/4EBP1 signaling could provide maximal clinical benefit to patients with OV by inducing ferroptosis.

## Results

### MEK inhibitors trigger ferroptosis in OV.

**Figure 1:**
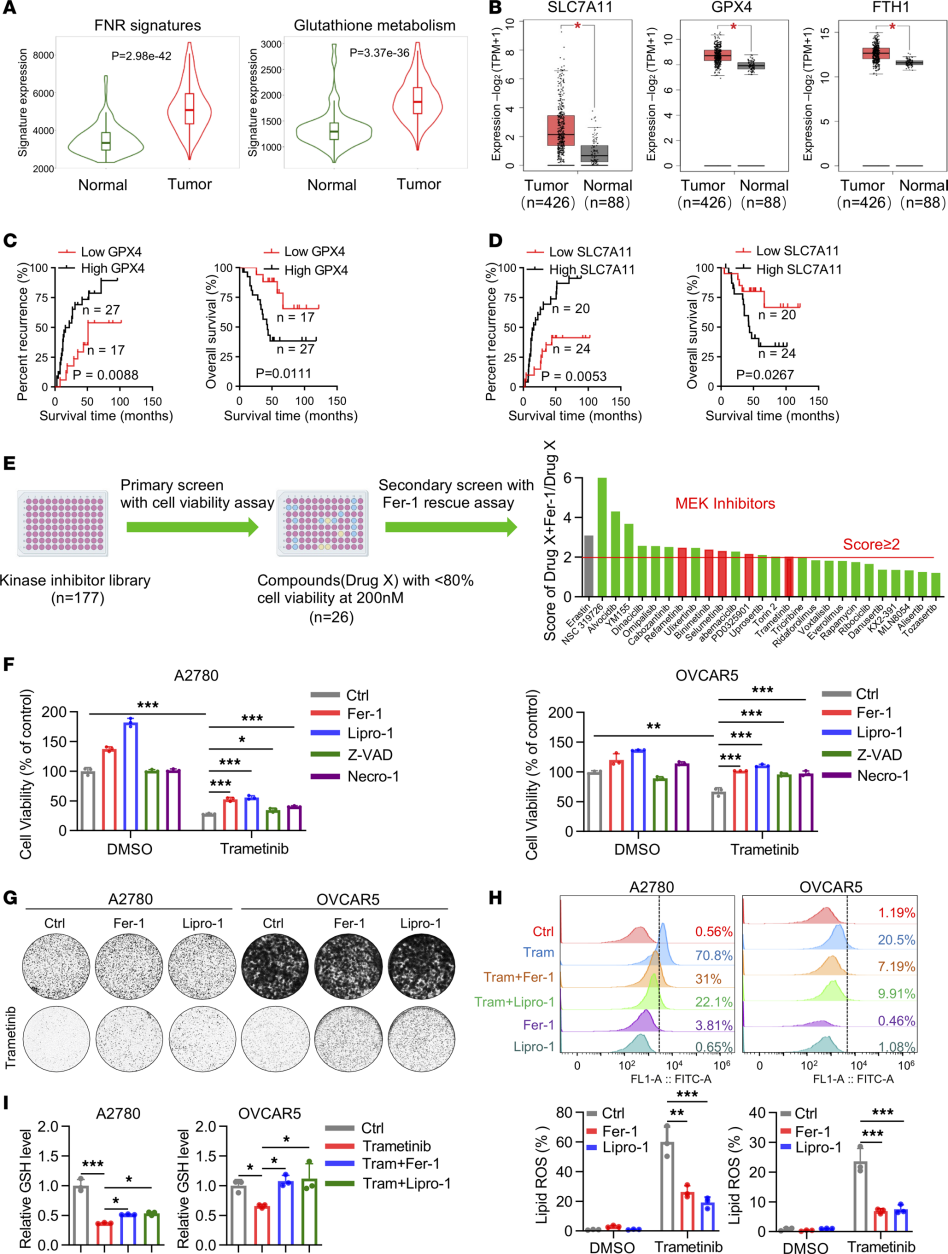
MEK inhibitors trigger ferroptosis in OV. (**A**) Gene expression levels of FNR signatures and glutathione metabolism pathway in OV tumor and normal tissues analyzed in TNMplot database. (**B**) Gene expression level of GPX4, SLC7A11, and FTH1 in TCGA OV tumor (*n* = 426) and matched TCGA normal OV tissues along with GTEx data (*n* = 88). (**C**) Kaplan-Meier curves of recurrence time and overall survival rates in patients with OV grouped according to high (black, *n* = 17) and low (red, *n* = 27) expression of GPX4. (**D**) Kaplan-Meier curves of recurrence time and overall survival rates in patients with OV grouped according to high (black, *n* = 20) and low (red, *n* = 24) expression of SLC7A11. (**E**) The screening process for discovering ferroptosis inducers by performing kinase inhibitor library screening with 177 compounds in A2780. (**F**) Cell viability assay of A2780 and OVCAR5 cells treated with vehicle (DMSO) or trametinib (200 nM in A2780 and 500 nM in OVCAR5) in the absence or presence of Ferrostatin-1 (Fer-1) (2 μM), Liproxstatin-1 (Lipro-1) (100 nM), Necrostatin-1 (Necro-1) (5 μM), and Z-VAD-FMK (Z-VAD) (5 μM) for 72 hours. (**G**) Colony formation assay in A2780 and OVCAR5 treated with vehicle (DMSO) or trametinib (100 nM in A2780 and 200 nM in OVCAR5) in the absence or presence of Fer-1 (2 μM) and Lipro-1 (100 nM). (**H**) Lipid peroxidation assay and (**I**) intracellular GSH level of A2780 and OVCAR5 treated with trametinib (200 nM in A2780 and 500 nM in OVCAR5) with or without Fer-1 or Lipro-1 for 48 hours. (**C** and **D**) *P* values were determined by log-rank test. (**F**, **H** and **I**) Results are represented as mean ± SD of 3 biological replicates. *P* values were determined by 1-way ANOVA with Bonferroni’s post hoc test. *P < 0.05, **P < 0.01, ***P < 0.001.

Ferroptosis-related genes are characterized as ferroptosis positive regulation signatures or FNR signatures in the FerrDb database (14). To determine whether ferroptosis escape plays an important role in OV, we first examined the expression of FNR signatures and glutathione metabolism pathway by TNM plot analysis (15), both of which were found to be significantly upregulated in OV compared with healthy ovarian tissues (Figure 1A). The Cancer Genome Atlas (TCGA) and Genotype-Tissue Expression (GTEx) database profiling analysis showed that expressions of ferroptosis suppressors, including SLCA711, GPX4, and FTH1, were also significantly increased in OV compared with healthy ovarian tissues (Figure 1B). We further verified the increased protein level by immunohistochemistry (IHC) analysis in OV tissues. Our analysis showed that high expression of both SLC7A11 and GPX4 was associated with poor patient outcomes (Figure 1, C and D, and Supplemental Figure 1A; supplemental material available online with this article; https://doi.org/10.1172/jci.insight.177857DS1). These data suggest that loss of ferroptosis is associated with the tumorigenesis and development of OV, indicating that reactivation of ferroptosis may be an effective therapeutic option for patients with OV.

To identify novel ferroptosis inducers, we performed a kinase inhibitor library screening with 177 kinase inhibitors in OV cell line A2780. Twenty-six drugs were selected as their inhibition rate of cell viability was more than 20%. We next performed a secondary screening by combining the selected drugs with the ferroptosis inhibitor Ferrostatin-1 (Fer-1). We identified 15 drugs with a combo/Fer-1 ratio of more than 2 as potential ferroptosis inducers (Figure 1E), with erastin used as a positive control of ferroptosis inducer (Supplemental Figure 1, B and C). Interestingly, 5 MEK inhibitors were among these drugs (Figure 1E). Verifying that ferroptosis is the cell death pathway involved, further studies showed that one of the MEK inhibitors, trametinib, could remarkably inhibit cell proliferation, and the inhibitory effect was significantly rescued by ferroptosis inhibitors, Fer-1 and Liproxstatin-1 (Lipro-1). In contrast, the apoptosis inhibitor Z-VAD-FMK (Z-VAD) and the necroptosis inhibitor Necrostatin-1 (Necro-1) could only partially rescue the inhibitory effect of trametinib (Figure 1, F and G, and Supplemental Figure 1D). We next detected ferroptosis by determining the amount of lipid peroxides in cellular membranes using BODIPY-C11 probe and flow cytometry analysis. The data showed that trametinib significantly induced lipid peroxidation in A2780 and OVCAR5, which could be significantly reversed by Fer-1 and Lipro-1 (Figure 1H). Moreover, trametinib significantly decreased GSH levels, which were partially restored by both Fer-1 and Lipro-1 (Figure 1I). We verified the above findings with another MEK inhibitor, PD0325901 (Supplemental Figure 1, E and F). Taken together, these findings suggested that MEK inhibitors could induce ferroptosis and offer an alternative therapy for patients with OV.

### Loss of ferroptosis is associated with resistance to MEK inhibitors in OV.

**Figure 2:**
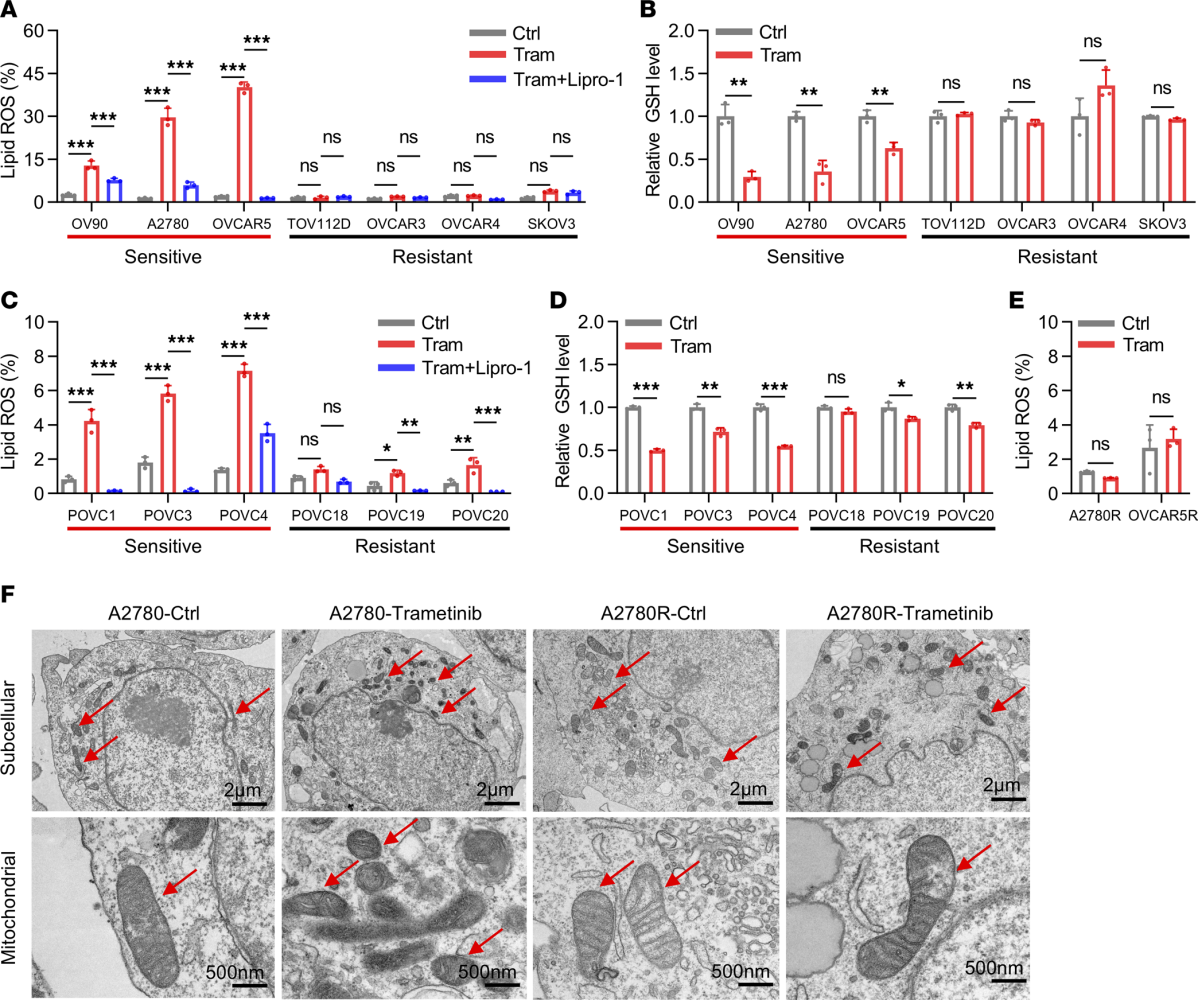
Loss of ferroptosis is associated with the resistance to MEK inhibitors in OV. (**A**) Lipid ROS level and (**B**) intracellular GSH level of commercial OV cell lines treated with trametinib (Tram, 200 nM) combined with or without Lipro-1 (100 nM). (**C**) Lipid ROS level and (**D**) intracellular GSH level of OV patient-derived cells (PDCs) treated with trametinib (200 nM) combined with or without Lipro-1 (100 nM). (**E**) Lipid peroxidation level of trametinib acquired-resistant cells A2780R and OVCAR5R treated with or without trametinib (200 nM). (**F**) A2780 and A2780R cells were treated with trametinib (200 nM) and analyzed by TEM to detect ultrastructure of mitochondria in 2 scale bars, 500 nm and 2 μm. The data are presented as the mean ± SD of three independent experiments. (**A** and **C**) *P* values were determined by 1-way ANOVA with Bonferroni’s post hoc test; (**B**, **D** and **E**) *P* values were determined by unpaired Student’s *t* test. *P < 0.05, **P < 0.01, ***P < 0.001.

To evaluate the effect of MEK inhibitors on inducing ferroptosis in different OV models, we evaluated lipid peroxidation and intracellular GSH levels in a panel of commercial OV cell lines and OV patient–derived cells (PDCs). To validate the sensitivity of trametinib in OV cell lines, we performed cell viability assay of A2780 and its acquired MEK inhibitor–resistant cell A2780R. The data showed that A2780 was sensitive to trametinib while A2780R was persistently resistant at the same dosage of trametinib (Supplemental Figure 2A). Furthermore, we performed cell viability assay of 7 commercial OV cell lines and 6 patient-derived primary cells with trametinib treatment. The results showed that OV90, A2780, OVCAR5, POVC1, POVC3, and POVC4 were MEK inhibitor sensitive while TOV112D, OVCAR3, OVCAR4, SKOV3, POVC18, POVC19, and POVC20 were MEK inhibitor resistant (Supplemental Figure 2, B and C). The results above are consistent with our previous study (16). Notably, trametinib could significantly trigger lipid peroxidation and decrease GSH level in both commercial OV cell lines and PDCs that are sensitive to MEK inhibitors. In contrast, trametinib could only slightly or modestly increase lipid ROS and suppress GSH level in resistant cells (Figure 2, A–D). In addition, by comparing the MEK inhibitor–sensitive cell lines A2780 and OVCAR5 with their respective acquired-resistant cell lines, A2780R and OVCAR5R, we observed that trametinib only slightly or barely induced lipid peroxidation in the resistant cell lines while markedly triggering lipid peroxidation in the sensitive cell lines (Figure 2E). Moreover, transmission electron microscopy (TEM) revealed significant differences in the ultrastructural analysis of mitochondria between the MEK inhibitor–sensitive (A2780) and –resistant (A2780R) cell lines (Figure 2F). Notably, after treatment with trametinib, A2780 exhibited shrunken mitochondria with elevated membrane density, a hallmark of ferroptosis, while A2780R did not, suggesting that ferroptosis might be triggered only in MEK inhibitor–sensitive cell lines. Collectively, these findings suggested that ferroptosis was more readily triggered in MEK inhibitor–sensitive cell lines and that escape of ferroptosis may contribute to the resistance of OV cells to MEK inhibitors. Therefore, reactivation of ferroptosis may represent a promising strategy to overcome MEK inhibitor resistance in OV.

### SLC7A11 protein synthesis dictates the sensitivity of OV cells to ferroptosis triggered by MEK inhibitors.

**Figure 3:**
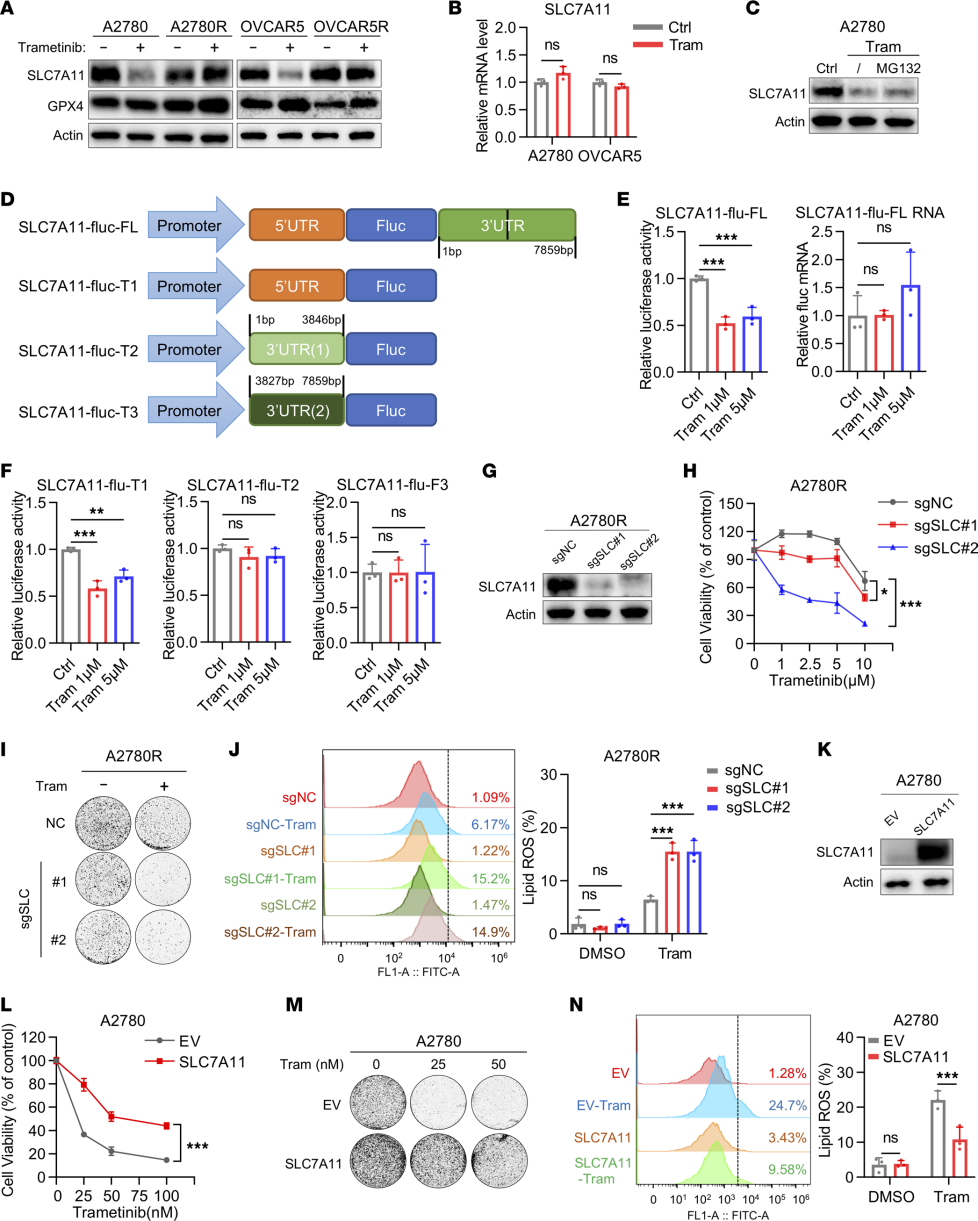
SLC7A11 protein synthesis dictates the sensitivity of OV cells to ferroptosis triggered by MEK inhibitors. (**A**) Immunoblot analysis of SLC7A11 and GPX4 in A2780 and OVCAR5 cells with their counterpart resistant lines treated with trametinib (200 nM) for 48 hours. (**B**) qRT-PCR analysis of *SLC7A11* in A2780 and OVCAR5 cells treated with trametinib (200 nM) for 48 hours. (**C**) Immunoblot analysis of SLC7A11 in A2780 cells treated with trametinib (200 nM) for 48 hours followed by 1.0 μM MG132 for 6 hours before harvest. (**D**) Patterns of SLC7A11 luciferase reporter plasmids. (**E**) Relative luciferase activity of SLC7A11- FL and the mRNA level of SLC7A11-FL tested by qRT-PCR in A2780 cells treated with trametinib for 48 hours. (**F**) Relative luciferase activity of SLC7A11-fluc-T1, SLC7A11-fluc-T2, and SLC7A11-fluc-T3 after transient transfection into A2780 cells. In **E** and **F**, data are represented as mean ± SD, *n* = 3. (**G**) The effect of CRISPR/Cas9-mediated SLC7A11 knockdown (sgSLC1 and sgSLC2) evaluated by immunoblot analysis. (**H**) Cell viability assay and (**I**) colony formation assay of the effect of SLC7A11 ablation on trametinib sensitivity. The concentration of trametinib used in colony formation assay is 10 μM. (**J**) The effect of SLC7A11 ablation on lipid peroxidation under trametinib treatment (10 μM). (**K**) The effect of SLC7A11 overexpression evaluated by immunoblot analysis in A2780. (**L**) Cell viability assay and (**M**) colony formation assay of empty vector (EV) and SLC7A11-overexpressing (SLC7A11) A2780 cells treated with trametinib. (**N**) Lipid peroxidation assay of A2780-EV and -SLC7A11 cells treated with trametinib (200 nM). Data are presented as the mean ± SD of triple independent experiments. *P* values were determined by (**B** and **N**) unpaired Student’s *t* test, (**E**, **F**, and **J**) 1-way ANOVA with Bonferroni’s post hoc test, or (**H** and **L**) 2-way ANOVA with Tukey’s post hoc test. *P < 0.05, **P < 0.01, ***P < 0.001.

Glutathione is a tripeptide synthesized from cysteine, glutamate, and glycine, with cysteine being the rate-limiting precursor. In most cancer cells, cysteine is acquired through the uptake of extracellular cystine via the amino acid transporter SLC7A11, which is then reduced to cysteine intracellularly, ultimately fueling GSH biosynthesis (6, 17–19). GPX4, a glutathione peroxidase, uses reduced GSH as a cofactor to suppress lipid peroxidation (5). Since trametinib decreased GSH levels in sensitive cells but not in resistant cell lines, we hypothesized that the SLCA711/GPX4 axis was associated with the sensitivity of OV cells to ferroptosis induced by MEK inhibitors. We examined the protein levels of SLC7A11 and GPX4, both of which are in the core signaling pathway of ferroptosis, in both MEK inhibitor–sensitive and –resistant cells treated with trametinib. Immunoblotting analysis showed that trametinib dramatically suppressed the expression of SLC7A11 in sensitive cells but not in resistant cells, while the level of GPX4 did not change in both sensitive and resistant cells (Figure 3A). Interestingly, mRNA level of *SLC7A11* was not suppressed by trametinib in sensitive cells, as shown by real-time quantitative reverse transcriptase PCR (qRT-PCR) (Figure 3B). Reduction of SLC7A11 protein levels without a corresponding decrease in its mRNA levels implies either increased protein degradation or decreased protein synthesis (or both) may contribute to SLC7A11 suppression upon trametinib treatment in sensitive cells. Treatment with the proteasome inhibitor MG132 did not rescue SLC7A11 protein levels under trametinib treatment (Figure 3C). Therefore, it is less likely that the change in SLC7A11 protein levels in response to trametinib treatment results from altered SLC7A11 protein degradation.

We next hypothesized that trametinib would regulate the protein synthesis of SLC7A11. To test this, we used 4 translation reporters, the promoter and different UTR fragments from *SLC7A11*: SLC7A11-fluc-FL (promoter region, 5′-UTR, and 3′-UTR), SLC7A11-fluc-T1 (promoter region and 5′-UTR), SLC7A11-fluc-T2 (promoter region, 5′-UTR, and nt 1–3,846 of 3′-UTR), and SLC7A11-fluc-T3 (promoter region, 5′-UTR, and nt 3,827–7,859 of 3′-UTR) (20) (Figure 3D). Using luciferase reporter assays, we found that the activity of luciferase reporter SLC7A11-fluc-FL was significantly inhibited by trametinib in A2780, whereas the mRNA level of SLC7A11-fluc-FL was not affected (Figure 3E). Moreover, only the activity of luciferase reporter SLC7A11-fluc-T1, but not that of SLC7A11-fluc-T2 or SLC7A11-fluc-T3, was significantly suppressed in A2780 treated with trametinib (Figure 3F). Our data suggested that trametinib regulated the translation of SLC7A11 protein through the first half of its 5′-UTR (the T1 region) in the sensitive cells. To further functionally validate that constitutive SLC7A11 activation contributed to resistance to ferroptosis induced by MEK inhibitors, SLC7A11 was depleted in A2780R cells using CRISPR/Cas9 method (Figure 3G). SLC7A11 depletion restored trametinib-induced lipid peroxidation and ferroptosis, as demonstrated by colony formation assay, ATP assay, propidium iodide assay, and lipid peroxidation assay (Figure 3, H–J, and Supplemental Figure 3, A–C). More importantly, overexpression of SLC7A11 significantly inhibited trametinib-triggered lipid peroxidation and ferroptosis in sensitive cells (Figure 3, K–N, and Supplemental Figure 3, D–F). These findings suggested that SLC7A11 dictates the sensitivity of OV cells to ferroptosis induced by MEK inhibitors.

### mTOR/4EBP1 pathway modulates SLC7A11 protein synthesis to promote ferroptosis escape upon trametinib treatment.

**Figure 4:**
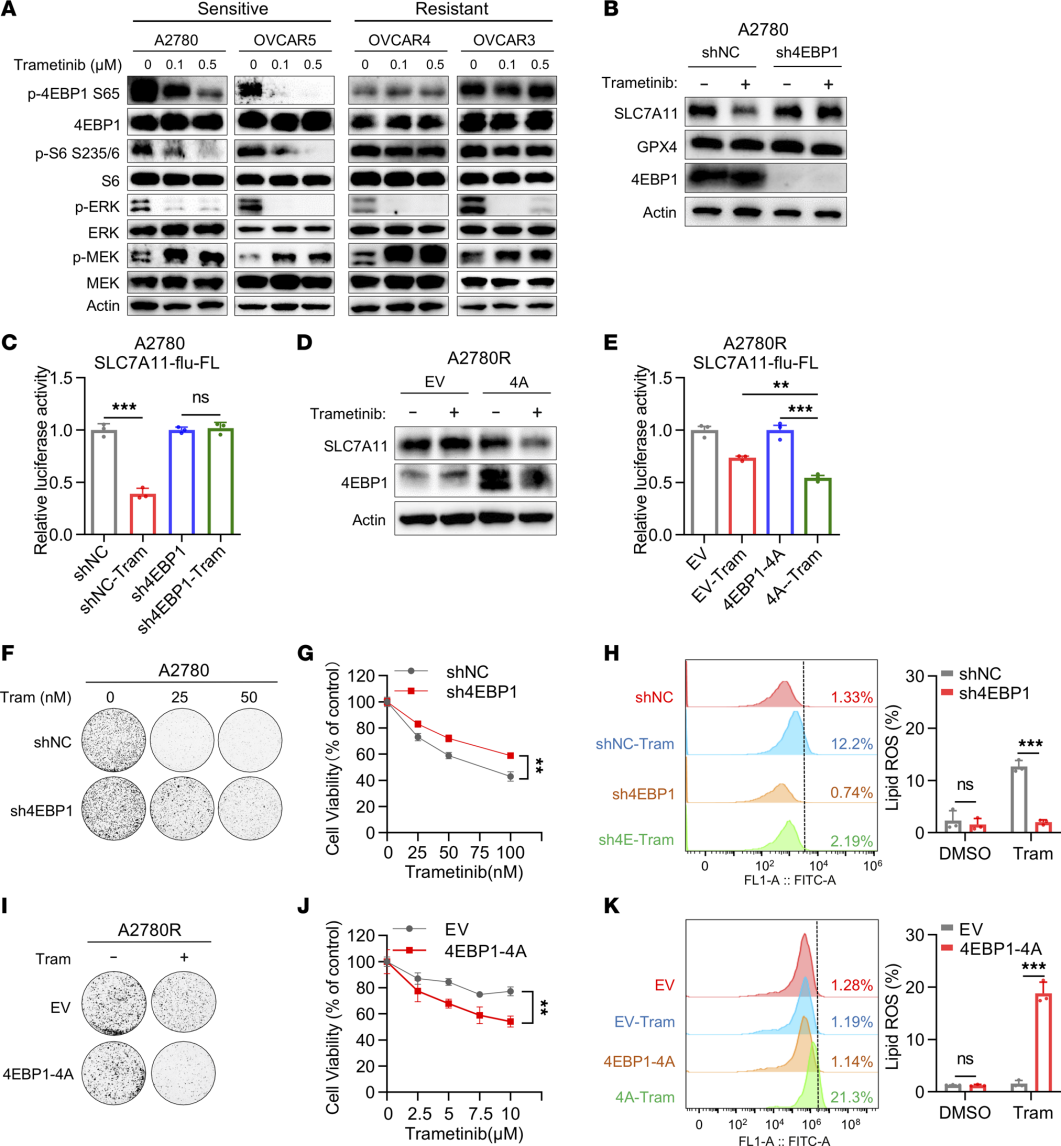
mTOR/4EBP1 pathway modulates SLC7A11 protein synthesis to promote ferroptosis escape upon trametinib treatment. (**A**) Immunoblot analysis of AKT, 4EBP1, S6, and ERK and MEK activity in A2780, OVCAR5, OVCAR3, and OVCAR4 cells treated with vehicle, 100 nM trametinib, or 500 nM trametinib. p-, phosphorylated. (**B**) Immunoblot analysis of SLC7A11, GPX4, and 4EBP1 in A2780 treated with trametinib (200 nM) after transfection with either negative control (shNC) or sh4EBP1. (**C**) The relative luciferase activity of SLC7A11-flu-FL in A2780 treated with trametinib after transfection with either negative shNC or sh4EBP1. (**D**) Immunoblot analysis of SLC7A11, GPX4, and 4EBP1 in A2780R cells treated with trametinib (10 μM) after stable expression of either EV or 4EBP1-4A. (**E**) The relative luciferase activity of SLC7A11-flu-FL in A2780R treated with trametinib after transfection with either EV or 4EBP1-4A. (**F**) Colony formation assay and (**G**) cell viability assay of the effect of 4EBP1 depletion on trametinib sensitivity. (**H**) The effect of 4EBP1 depletion on lipid peroxidation in A2780 treated with trametinib (200 nM). (**I**) Colony formation assay and (**J**) cell viability assay of the effect of 4EBP1-4A overexpression on trametinib sensitivity (trametinib, 10 μM). (**K**) The effect of 4EBP1-4A overexpression on lipid peroxidation in A2780R treated with trametinib (10 μM). The data are presented as the mean ± SD of 3 independent experiments. (**C**, **H**, and **K**) *P* values were determined by unpaired Student’s *t* test. (**E**) *P* values were determined by 1-way ANOVA with Bonferroni’s post hoc test. (**G** and **J**) Two-way ANOVA with Tukey’s post hoc test. **P < 0.01, ***P < 0.001.

mTOR signaling pathway is associated with protein translation (21–23). The major effects of mTOR on translation are mediated by its phosphorylation of 4EBPs. Dephosphorylated 4EBPs bind to cap-binding protein eIF4E to interfere with the assembly of the preinitiation complex (24–26). Phosphorylation of 4EBPs by mTOR releases 4EBPs from eIF4E, thereby allowing 5′-cap–dependent translation initiation (27). To determine whether MEK inhibitors regulate the mTOR/4EBP1 pathway, we examined the activity of mTOR/4EBP1 pathway upon trametinib treatment by immunoblotting analysis. The data showed that trametinib caused a remarkable decrease in 4EBP1 and S6K phosphorylation in sensitive cells while resistant cells maintained persistent 4EBP1 and S6K activation (Figure 4A and Supplemental Figure 4A), which was consistent with decreased SLC7A11 level after trametinib treatment in sensitive cells. We next investigated whether the mTOR/4EBP1 axis is involved in trametinib-mediated SLC7A11 inhibition by deleting 4EBP1 from A2780 with shRNA. In contrast with control group (shNC), MEK inhibitors did not decrease the expression of SLC7A11 in MEK inhibitor–sensitive cell A2780 with 4EBP1 knocked down (Figure 4B). Further luciferase reporter assays manifested that knockdown of 4EBP1 reversed the suppressive effect of trametinib on SLC7A11 translation in A2780 (Figure 4C). These data indicate that trametinib-mediated SLC7A11 inhibition depends on the activity of the mTOR/4EBP1 axis.

4EBP1-4A is a nonphosphorylatable mutant of 4EBP1, in which its phosphorylation sites are replaced with alanines (T37, T46, S65, and T70), allowing 4EBP1 to bind to eIF4E constitutively and inhibit cap-dependent translation (28, 29). Ectopic expression of 4EBP1-4A could suppress the protein level of SLC7A11 in resistant A2780R cells upon trametinib treatment (Figure 4D). Luciferase reporter assays showed that ectopic expression of 4EBP1-4A enhanced the inhibitory effect of trametinib on SLC7A11 translation in A2780R (Figure 4E). These data revealed that mTOR1/4EBP1 modulated SLC7A11 protein synthesis upon trametinib treatment.

To further determine whether 4EBP1 could modulate the sensitivity to ferroptosis induced by trametinib, we conducted shRNA-mediated 4EBP1 depletion. We found that 4EBP1 depletion could impair trametinib sensitivity. 4EBP1-depleted A2780 treated with trametinib resulted in more colonies and higher cell viability, as well as less lipid ROS production (Figure 4, F–H). Moreover, ectopic expression of 4EBP1-4A resensitized the resistant A2780R cells to trametinib-induced ferroptosis as evidenced by lower colony formation, lower cell viability, as well as enhanced lipid peroxidation (Figure 4, I–K). Together, our findings suggested that sustained mTOR1-4EBP1 activity maintained SLC7A11 translation, mediating ferroptosis escape upon trametinib treatment in OV.

### Targeting PI3K/mTOR signaling sensitizes resistant cells to ferroptosis induced by MEK inhibitors.

**Figure 5:**
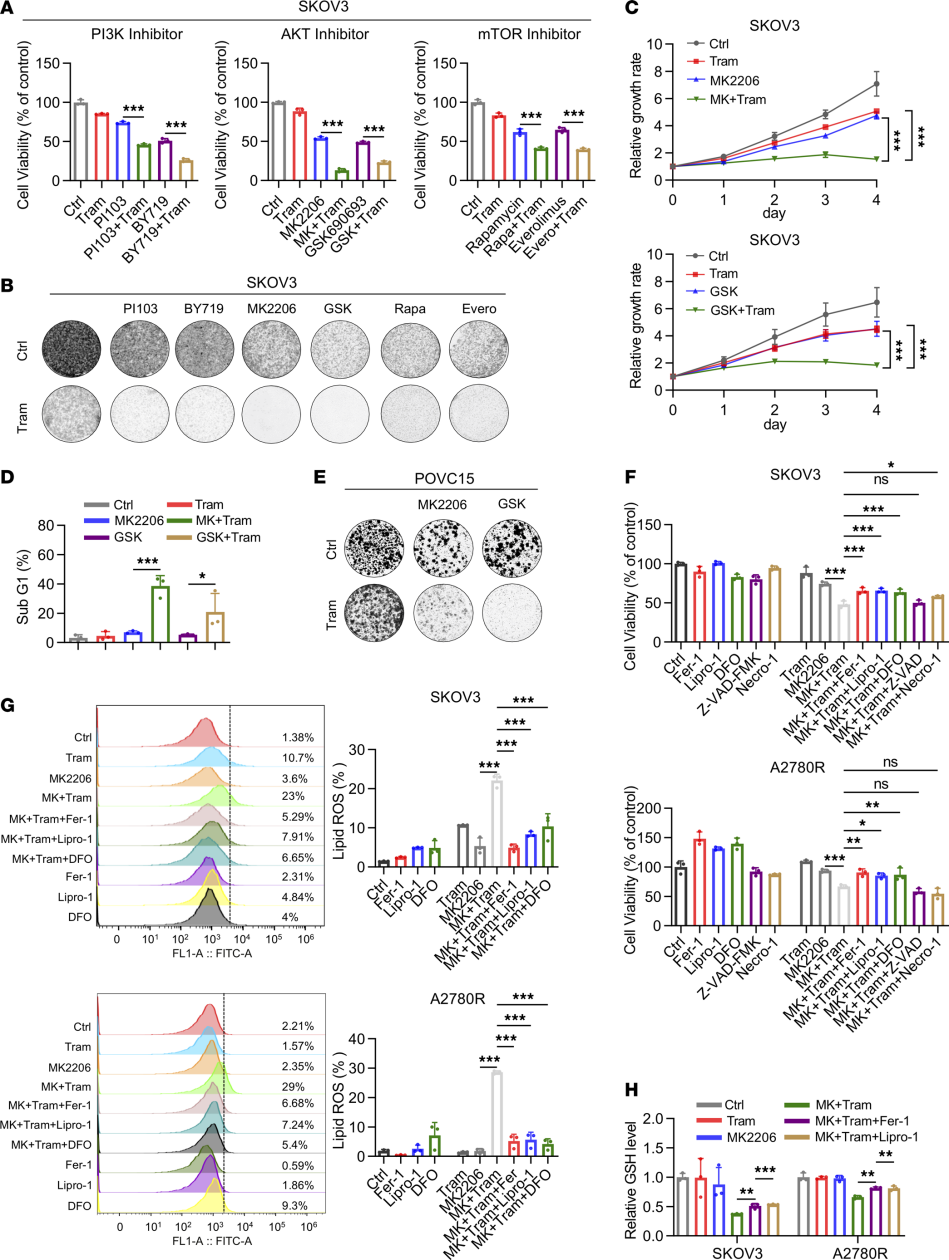
Targeting PI3K/mTOR signaling sensitizes resistant cells to ferroptosis induced by MEK inhibitors. (**A**) Cell viability of SKOV3 treated with 500 nM trametinib with or without PI3K/AKT/mTOR inhibitors for 96 hours. BY719 (1 μM), PI103 (1 μM), MK2206 (MK) (5 μM), GSK690693 (GSK) (5 μM), rapamycin (Rapa) (1 μM), everolimus (Evero) (0.5 μM). (**B**) Colony formation of SKOV3 treated with 500 nM trametinib with or without PI3K/AKT/mTOR inhibitors. BY719 (1 μM), PI103 (1 μM), MK2206 (MK) (5 μM), GSK690693 (GSK) (5 μM), rapamycin (Rapa) (1 μM), everolimus (Evero) (0.5 μM). (**C**) Growth curves and (**D**) sub-G_1_ population analysis in SKOV3 treated with vehicle, trametinib, AKT inhibitors (GSK690693 or MK2206), or their combination. (**E**) Colony formation assay of PDC-POVC15 treated with trametinib (100 nM) with or without AKT inhibitors (MK2206, 5 μM and GSK690693, 5 μM). (**F**) Cell viability of SKOV3 and A2780R cells following trametinib (500 nM) or MK2206 treatment (5 μM) in the presence or absence of Fer-1 (2 μM), Lipro-1 (100 nM), DFO (300 nM), Z-VAD (5 μM), and Necro-1 (5 μM) for 48 hours. (**G**) Detection of lipid peroxidation level with BODIPY 581/591 C11 probe determined by the flow cytometer in SKOV3 and A2780R treated with trametinib (500 nM) or MK2206 (5 μM) treatment in the presence or absence of Fer-1, Lipro-1, and DFO for 48 hours. (**H**) Detection of GSH level in SKOV3 and A2780R followed by trametinib (500 nM) or MK2206 treatment (5 μM) in the presence or absence of Fer-1 or Lipro-1 for 48 hours. The data are presented as the mean ± SD of 3 independent experiments. (**A** and **F**–**H**) *P* values were determined by 1-way ANOVA with Bonferroni’s post hoc test. (**C**) *P* values were determined by 2-way ANOVA with Tukey’s post hoc test. (**D**) *P* values were determined by unpaired Student’s *t* test. *P < 0.05, **P < 0.01, ***P < 0.001.

To sensitize resistant cells to ferroptosis induced by MEK inhibitors, we sought to explore an approach that targets protein synthesis and induces ferroptosis. Since the PI3K/AKT/mTOR signaling pathway is a key regulator of protein translation (30), we investigated the combination of inhibitors targeting this pathway with trametinib. We found that combined treatment of PI3K/AKT/mTOR inhibitors and trametinib had a combinatorial effect in suppressing cell proliferation in SKOV3 and A2780R cells (Figure 5, A and B, and Supplemental Figure 5, A and B). Further experiments showed that combined treatment of AKT inhibitors and trametinib had a combinatorial effect in inducing cell death and suppressing cell proliferation in both intrinsically resistant and acquired-resistant cells (Figure 5, C–E, and Supplemental Figure 5C). To investigate whether ferroptosis was involved in this effect, we treated SKOV3 and A2780R cells with MK2206 (AKT inhibitor) and trametinib in the presence or absence of ferroptosis rescue agents. We observed that cell proliferation inhibited by the combination of MK2206 and trametinib was partially restored by the ferroptosis inhibitors deferoxamine (DFO), Lipro-1, or Fer-1 (Figure 5F). Moreover, we detected several ferroptotic events, including lipid peroxidation accumulation and GSH depletion in SKOV3 and A2780R cells. Following treatment with the combination of MK2206 and trametinib, lipid ROS accumulation was significantly increased, and this effect was partially impaired by Lipro-1 and Fer-1 (Figure 5G). Meanwhile, GSH levels were significantly reduced by the treatment of MK2206 and trametinib, indicating GSH depletion had occurred (Figure 5H). These results suggested that targeting PI3K/mTOR signaling can sensitize resistant cells to ferroptosis induced by trametinib.

### Cotargeting AKT and MEK suppresses the protein synthesis of SLC7A11 via inhibition of mTOR/4EBP1 activity.

**Figure 6:**
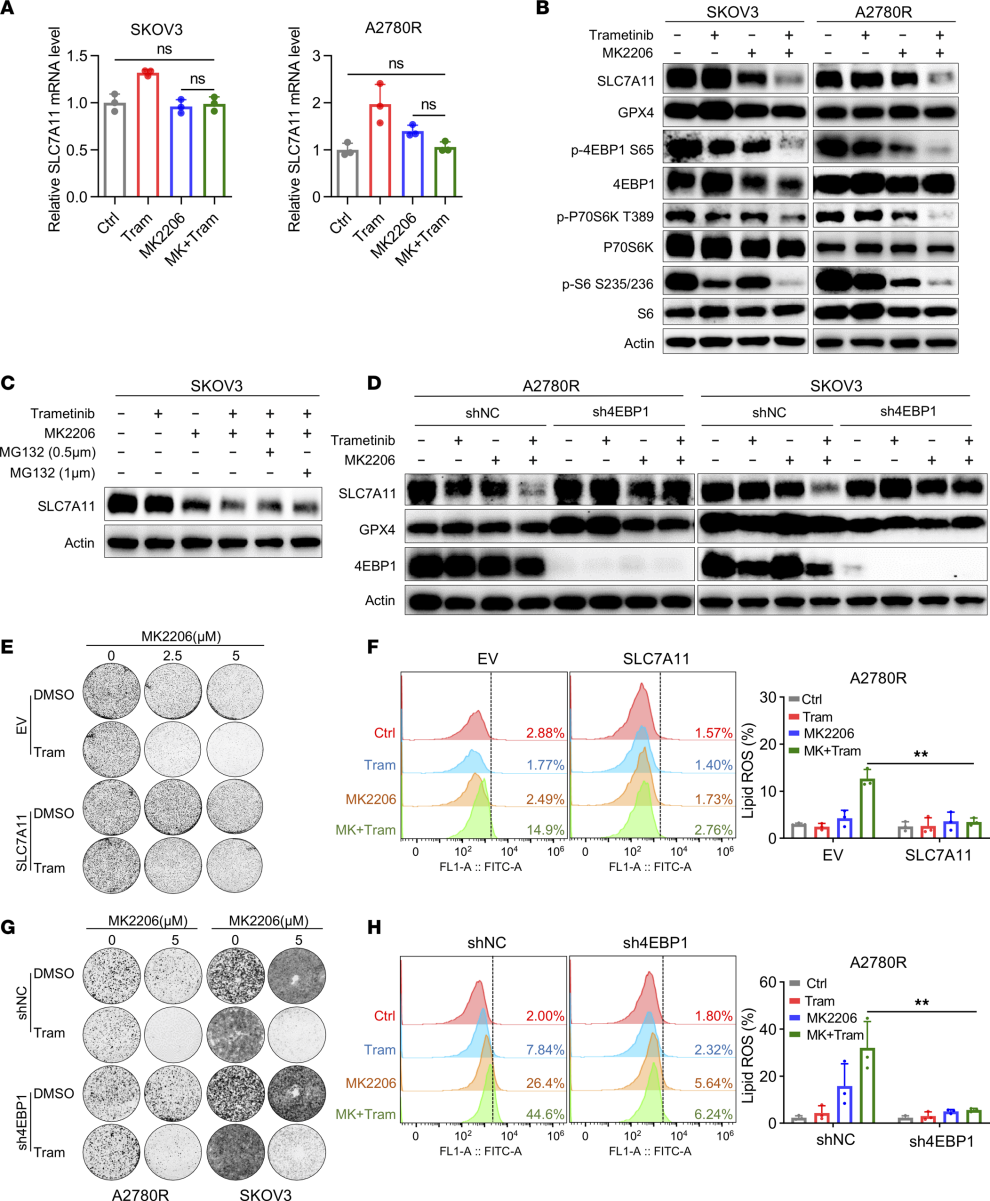
Cotargeting AKT and MEK suppresses the protein synthesis of SLC7A11 via inhibition of mTOR/4EBP1 activity. (**A**) The mRNA level of SLC7A11 in SKOV3 and A2780R treated with vehicle, trametinib (500 nM), MK2206 (5 μM), or their combination for 48 hours. (**B**) Immunoblot analysis of SLC7A11 and GPX4 and the activity of mTOR, 4EBP1, P70S6K, and S6 in SKOV3 and A2780R cells treated with vehicle, trametinib (500 nM), MK2206 (5 μM), or their combination for 48 hours. (**C**) Immunoblot analysis of SLC7A11 in SKOV3 treated with trametinib or MK2206 treatment for 48 hours in the presence or absence of MG132 at indicated concentrations for 6 hours before harvest. (**D**) Immunoblot analysis of SLC7A11 in SKOV3 and A2780R treated with trametinib (500 nM) or MK2206 (5 μM) after transfection with either shNC or sh4EBP1 for 48 hours. (**E**) Representative images of colony formation assay in A2780R cells treated with trametinib (500 nM) with or without MK2206 (both 2.5 μM and 5 μM) after transfection with either EV or SLC7A11. (**F**) Lipid peroxidation analysis in A2780R cells treated with trametinib (500 nM) with or without MK2206 (5 μM) for 48 hours after transfection with either EV or SLC7A11. (**G**) Representative images of colony formation assay in A2780R and SKOV3 cells treated with trametinib (500 nM) with or without MK2206 (5 μM) after transfection with either shNC or sh4EBP1. (**H**) Lipid peroxidation analysis in A2780R cells treated with trametinib (500 nM) with or without MK2206 (5 μM) for 48 hours after transfection with either shNC or sh4EBP1. The data are presented as the mean ± SD of 3 independent experiments. (**A**) *P* values were determined by 1-way ANOVA with Bonferroni’s post hoc test. (**F** and **H**) *P* values were determined by unpaired Student’s *t* test. **P < 0.01.

To investigate whether targeting PI3K/mTOR could sensitize resistant cells to trametinib-induced ferroptosis by suppressing SLC7A11 protein levels, we performed immunoblot analysis and qRT-PCR. As expected, the mRNA level of *SLC7A11* was not affected under the treatment of AKT inhibitor and trametinib, while the protein level of SLC7A11 was markedly inhibited in both SKOV3 and A2780R (Figure 6, A and B). More importantly, treatment with the proteasome inhibitor MG132 did not restore SLC7A11 protein levels under combination treatment (Figure 6C). The above data suggested that it is more likely that the change in SLC7A11 protein levels in response to cotreatment results from altered SLC7A11 protein synthesis than protein degradation.

A previous study has shown that combined inhibition of AKT and MEK kinase can cause the recruitment of 4EBP1 to suppress cap-dependent translation (28). Our data also verified that combined inhibition of MEK and AKT inhibited phosphorylation of important downstream mTOR signaling molecules, p70S6K, S6, and 4EBP1, without any effect on mTOR, MEK, or ERK phosphorylation in trametinib-resistant OV cells (Figure 6B and Supplemental Figure 6A). In addition, combined treatment of mTOR inhibitor rapamycin with MEK inhibitor could also inhibit phosphorylation of 4EBP1 and consequently suppress SLC7A11 protein level (Supplemental Figure 6B), suggesting that mTOR/4EBP1 activity was associated with the synthesis of SLC7A11. To investigate whether 4EBP1 is involved in the synthesis of SLC7A11 in the combination of MEK and AKT inhibitors, we conducted shRNA-mediated knockdown of 4EBP1 in A2780R and SKOV3. The results showed that compared with shNC group, SLC7A11 was restored by 4EBP1 depletion under the treatment of trametinib and MK2206 (Figure 6D). The above findings suggested that cotargeting AKT and MEK suppressed the protein synthesis of SLC7A11 dependent on 4EBP1. To further functionally test the role of the 4EBP1/SLC7A11 axis in regulating ferroptosis caused by MK2206 and trametinib, we first overexpressed SLC7A11 in A2780R by lentivirus infection (Supplemental Figure 6C). The results showed that SLC7A11 ectopic expression substantially impaired the combination effect of colony formation (Figure 6E). Furthermore, overexpression of SLC7A11 obviously reduced lipid peroxidation induced by treatment with MK2206 and trametinib in A2780R (Figure 6F). Likewise, 4EBP1 knockdown significantly impaired the combination effect and partially restored the lipid ROS induced by the combination (Figure 6, G and H). Collectively, these results indicated that AKT inhibitor MK2206 could restrain the protein synthesis of SLC7A11 dependent on 4EBP1 to sensitize resistant cells to trametinib-induced ferroptosis in OV.

### AKT inhibitor sensitizes OV to MEK inhibitor–mediated ferroptosis in vivo.

**Figure 7:**
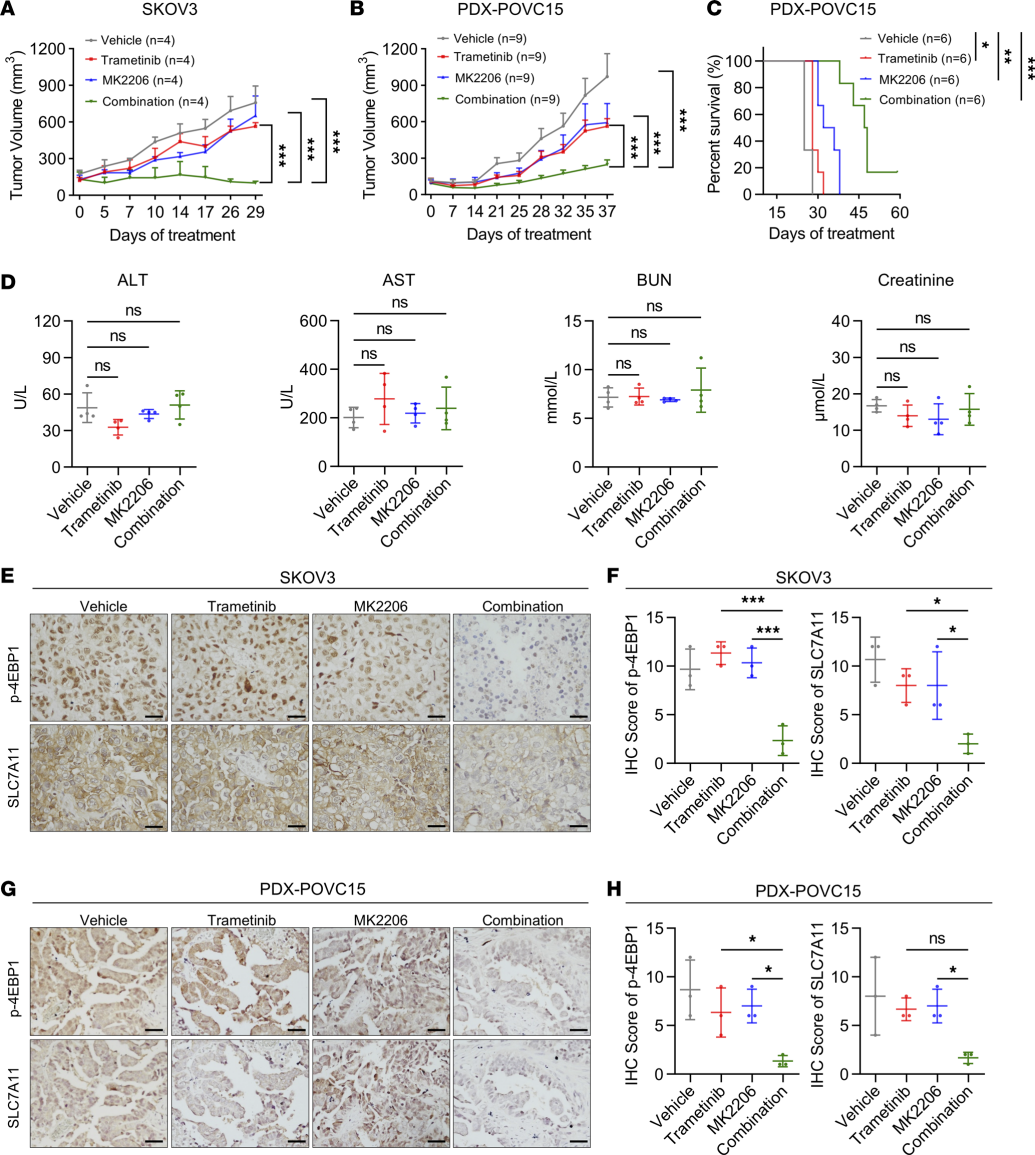
AKT inhibitor sensitizes OV to MEK inhibitor–mediated ferroptosis in vivo. (**A**) Tumor volume of SKOV3 xenografts in nude mice treated with vehicle, 60 mg/kg MK2206 (orally), 0.25 mg/kg trametinib (i.p.), or the combination at the same doses every other day (*n* = 4 per group). (**B**) Tumor volume of patient-derived xenograft PDX-POVC15 tumors implanted into NOD-SCID mice treated with vehicle, 90 mg/kg MK2206 (orally), 0.25 mg/kg trametinib (i.p.), or the combination at the same doses every other day (*n* = 9 per group). (**C**) Survival rates of patient-derived xenograft PDX-POVC15 tumors implanted into NOD/SCID mice treated with vehicle, 90 mg/kg MK2206 (orally), 0.25 mg/kg trametinib (i.p.), or the combination at the same doses every other day (*n* = 6 per group). The curve represents the survival time from the beginning of therapy. Drug treatment was withdrawn until the tumor volume of the first mouse reached 1,000 mm^3^ at day 25. (**D**) Quantification of ALT, AST, BUN, and creatinine levels in the serum of PDX-POVC15 of experiments described in **C** at day 25 (*n* = 4 per group). (**E**) Representative IHC and (**F**) quantification of p-4EBP1 and SLC7A11 in SKOV3 of experiments described in **A**. Scale bar, 50 μm. (**G**) Representative IHC and (**H**) quantification of p-4EBP1 and SLC7A11 in PDX-POVC15 of experiments described in **B**. Scale bar, 100 μm. (**A**–**C**) Date are presented as mean ± SEM. *P* values were determined by 2-way ANOVA with Tukey’s post hoc test. (**D**, **F**, and **H**) Quantification is shown from 3 tumors. The data are presented as the mean ± SD. *P* values were determined by 1-way ANOVA with Bonferroni’s post hoc test. *P < 0.05, **P < 0.01, ***P < 0.001.

To investigate the potential of the combination of trametinib and MK2206 in vivo, we assessed the efficacy of this combinatorial therapy in xenograft tumor models. In the SKOV3 xenograft model and OV patient–derived xenograft (PDX) model PDX-POVC15, we observed that the combined treatment of trametinib and MK2206 resulted in a significant reduction in tumor growth and tumor weight compared with single-drug treatment (Figure 7, A and B, and Supplemental Figure 7A). In addition, the combined treatment of trametinib and MK2206 resulted in higher survival rate with a modest change in body weight in the PDX-POVC15 model (Figure 7C and Supplemental Figure 7B). We also detected the levels of alanine aminotransferase (ALT), aspartate aminotransferase (AST), blood urea nitrogen (BUN), and creatinine in each group. The results showed that there was no significant change in liver and kidney functions, indicating tolerable side effects for the combination treatment in PDX models (Figure 7D). We also performed IHC staining for SLC7A11 and p-4EBP1 (Ser65) in xenograft tumor samples. The IHC data showed that the combination of trametinib and MK2206 exhibited a remarkable suppression of SLC7A11 and p-4EBP1 (Figure 7, E–H). Taken together, these findings provided strong evidence that the combination of MEK inhibitor and AKT inhibitor may have a potent antitumor effect in OV treatment with tolerable side effects.

## Discussion

Despite advances in biological understanding and modern oncologic treatments of OV, it remains the most lethal amongst gynecological malignancies in women, with estimated survival rates of less than 30% in advanced stages (2). Currently, targeted drugs such as PARP1 and VEGF-A inhibitors are used to delay OV progression and improve survival rates, but response rates are typically less than 50% in the relapsed or refractory setting, and acquired drug resistance invariably occurs with prolonged usage (31). Therefore, other drugs that leverage the other vulnerabilities of OV, such as ferroptosis inducers, are being explored (11, 32). In addition, platinum-tolerant OV cells with altered glutathione metabolism that depend on GPX4 for survival have been shown to be highly susceptible to ferroptosis inducers, such as GPX4 inhibitors (33). Thus, there is an urgent need to explore new therapeutic strategies targeting ferroptosis for OV treatment to improve clinical outcomes.

**Figure 8:**
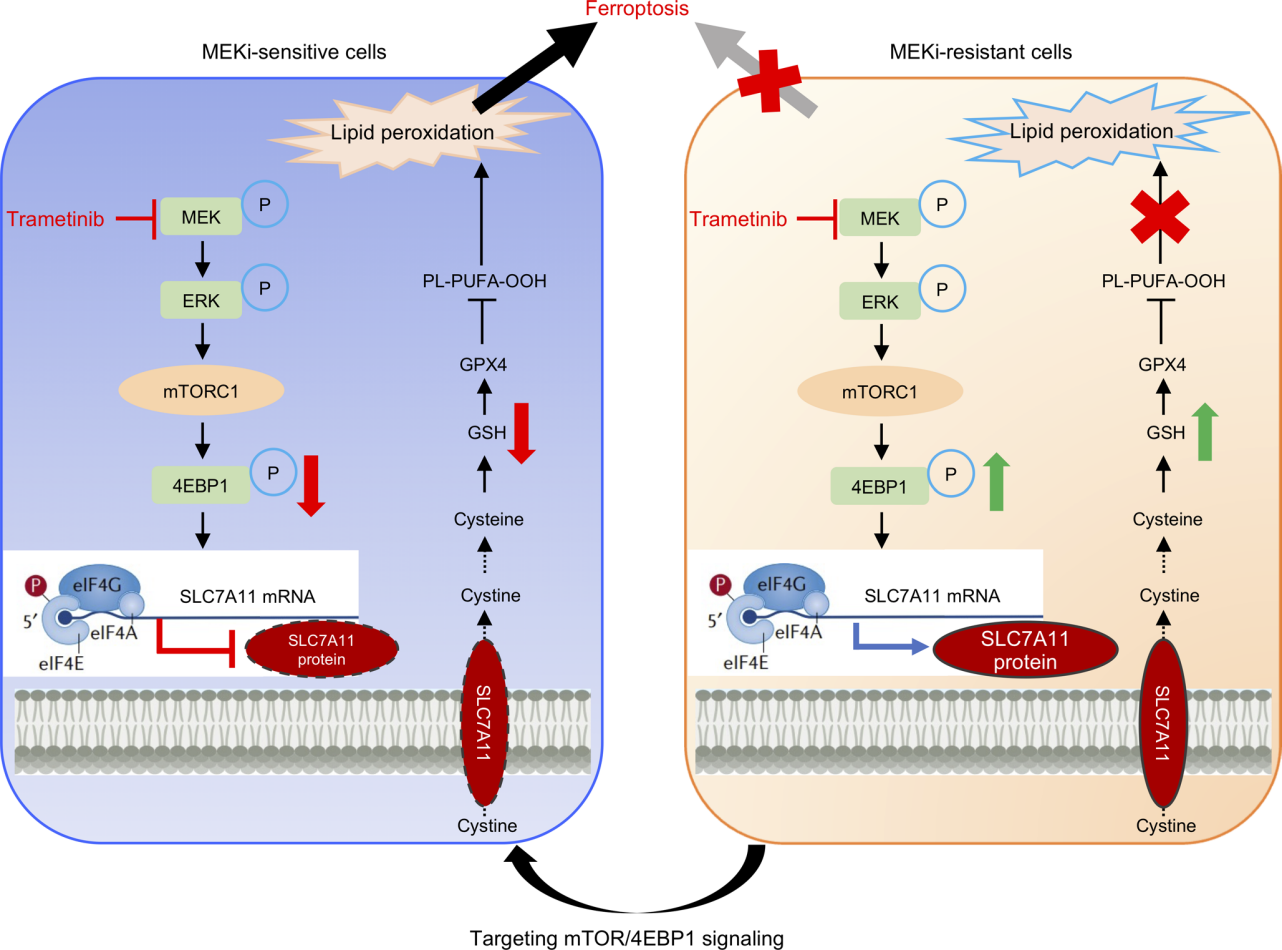
The schematic model illustrating the mechanism of ferroptosis modulated by MEK inhibitor. Trametinib inhibits mTOR/4EBP1 activity to suppress SLC7A11 protein synthesis, leading to ferroptosis in MEK inhibitor–sensitive OV cells (Left). Sustained mTOR/4EBP1 axis mediated SLC7A11 translation and conferred resistance to trametinib-induced ferroptosis (Right). Targeting mTOR/4EBP1 signaling reversed the resistance to ferroptosis induced by MEK inhibitors through suppression of SLC7A11 protein synthesis.

Targeting the MAPK signaling pathway has been explored across multiple studies in OV. In a recent meta-analysis on the clinical efficacy of monotherapy with a MAPK signaling pathway inhibitor, MEK inhibitors demonstrated a pooled overall response rate of 20% (34). In this study, we demonstrated the crucial role of FNR signatures in OV tumorigenesis and identified MEK inhibitors as potential inducers of ferroptosis in OV. However, we also found that certain groups of OV that are known to be resistant to MEK inhibitors are less susceptible to trametinib-induced ferroptosis, meaning that not all patients with OV will benefit from the treatment. Therefore, we embarked to mechanistically dissect how MEK inhibitor can induce ferroptosis, with the goal to improve effectiveness of clinical treatment that leverages the use of MEK inhibitor trametinib. We discovered that in MEK inhibitor–resistant OV cells, the protein synthesis of SLC7A11 was upregulated, which mediated the suppression of trametinib-induced ferroptosis. Interestingly, inhibition of mTOR/4EBP1 activity can repress the protein synthesis of SLC7A11 to promote trametinib-induced ferroptosis even in OV cells with resistance to MEK inhibitors (Figure 8), consistent with the roles of mTOR/4EBP1 in mRNA translation (25). The mTOR inhibitor rapamycin can decrease GPX4 protein translation at least partially by suppressing the activation of the Rag/mTOR/4EBP1 signaling axis (35). Another two studies also show that the synthesis of cyclin D1 and PTEN is regulated by mTOR-driven cap-dependent translation (36, 37). Therefore, therapeutically targeting mTOR/4EBP1/SLC7A11 axis is a viable option to promote trametinib-induced ferroptosis in OV.

The level of SLC7A11 could be regulated through multiple mechanisms, including transcriptional and posttranscriptional levels. For example, SLC7A11 could be transcriptionally upregulated by NRF2 (38). Deubiquitinase, such as OTUB1 and DUBA, could deubiquitinate and stabilize SLC7A11 protein to suppress ferroptosis (39, 40). In addition, another study reported that RBMS1 could bind to the eIF3d complex to promote SLC7A11 translation (20). Furthermore, SLC7A11 was found to be transcriptionally regulated by mTORC1 signaling via ATF4 (41). In our study, we demonstrated that mTOR signaling promotes SLC7A11 protein synthesis through 4EBP1 activity, whereas targeting mTOR/4EBP1 axis by MEK inhibitor could suppress SLC7A11 protein synthesis to induce ferroptosis.

Combination therapy with PI3K/AKT inhibitors and MAPK/ERK inhibitors has shown promise in preclinical studies by demonstrating synergistic antiproliferative activity in various cancers (42–44). However, combining AKT inhibitors with MEK inhibitors has shown tolerability issues in clinical trials (44, 45). Therefore, design and synthesis of new drugs that can cotarget MAPK and PI3K/AKT, to generate fewer adverse side effects, are critically warranted.

In conclusion, our study identifies a function of MEK inhibitors in triggering ferroptosis through suppression of the protein synthesis of SLC7A11. Sustained mTOR/4EBP1/SLC7A11 activity is associated with the resistance to ferroptosis induced by MEK inhibitors, but adding AKT inhibitors can overcome this resistance by inhibiting SLC7A11 protein synthesis. The efficacy of the combination approach has been proven in in vitro and in vivo OV models. Further investigation is needed to identify more effective therapeutic targets and minimize adverse side effects in the development of small molecule–targeted drugs for patients with OV. Ferroptosis has long been thought to increase the anticancer efficacy of immune checkpoint therapies (46, 47). Our proposed combination approach therefore also opens up avenues to synergize with immunotherapy to achieve greater anticancer effects for OV.

## Methods

### Sex as a biological variable.

These studies included only female animals and patients because OV is a disease that only occurs in assigned females at birth.

### Cell culture and reagents.

All commercial cell lines were purchased from ATCC, except for A2780 (obtained from the European Collection of Authenticated Cell Cultures). No authentication of cell lines was done by the authors. HEK293T cells were grown in DMEM (Gibco). A2780, OVCAR5, OV90, TOV112D, OVCAR3, OVCAR4, SKOV3, COV504, A2780R, and OVCAR5R cells were grown in RPMI-1640 (Gibco). PDCs were grown in DMEM/F12 (Gibco). Culture medium was supplemented with 10% bovine calf serum (Hyclone) and 1% penicillin/streptomycin (Gibco). Cells were confirmed to be cultured without mycoplasma. All cells were cultivated in 5% CO_2_ at 37°C. Other reagents were purchased as follows: trametinib (T2125), MK2206 (T1952), and GSK690693 (T6285) were purchased from Target Mol. Ferrostatin-1 (S7243), Liproxstatin-1 (S7699), Z-VAD-FMK (S7023), Necrostatin-1 (S8037), MG132 (S2619), PI103 (S1038), BY719 (S2814), rapamycin (S1039), everolimus (S1120), erastin (S7242), and kinase inhibitor drug library were purchased from Selleckchem.com. The drugs above were diluted in DMSO and stored at recommended conditions.

### Bioinformatics analysis.

FNR genes with score more than 2 were downloaded from FerrDb database V2 and analyzed in TNMplot database (https://tnmplot.com/analysis/) (15). Genes of glutathione metabolism were downloaded from Kyoto Encyclopedia of Genes and Genomes and analyzed in TNMplot database. A web server for cancer and normal gene expression profiling and interactive analyses, GEPIA (http://gepia.cancer-pku.cn/index.html), was used to determine the expression of SLC7A11, GPX4, and FTH1 in OV.

### Drug screening.

A2780 was subjected to a drug screen with a customized kinase inhibitor compound library. A total of 2,000 cells were seeded into a 96-well plate and treated with 186 compounds in the drug screen for 96 hours. Cell viability was assessed using CellTiter-Glo Luminescent Cell Viability Assay (G7570, Promega) according to the product instructions. Inhibitors that resulted in less than 80% cell viability in A2780 were selected for a secondary screen with Fer-1, a typical ferroptosis inhibitor, and drug scoring was calculated by dividing the score of Fer-1/selected drug combination by the single selected drug score. The drugs whose score was more than 2 were selected as potential ferroptosis inducers. Erastin was a positive control of inducing ferroptosis (48). The result of drug screening is listed in Supplemental Table 1.

### Cell viability and colony formation assay.

A total of 2,000 cells were seeded in a 96-well plate for 24 hours and treated with indicated drugs for 96 hours. Cell viability was measured by the CellTiter-Glo Luminescent Cell Viability Assay. Luminescence was detected by a Tecan Infinite M200 Pro plate reader. All conditions were replicated in triplicate. Drug curves were generated using GraphPad Prism 9.0 Software. For colony formation assay, 1 × 10^4^ cells were seeded in a 6-well plate and treated with indicated drugs for 9–12 days. The fresh medium was replaced every 3 days. After washing with PBS once, surviving colonies were fixed with methanol for 5 minutes and stained with crystal violet for 5 minutes.

### Propidium iodide staining and apoptosis assay.

Cell cycle analysis was done by propidium iodide staining (P4864, MilliporeSigma) to quantify the sub-G_1_ population, which can reflect the quantification of cell death. Briefly, 1 × 10^5^ cells were seeded in a 6-well plate and treated with indicated agents for 72 hours. Cells were harvested and fixed with 70% ethanol for at least 4 hours. The cells were then washed with PBS twice and stained with propidium iodide at a concentration of 50 mg/mL. For apoptotic assay, apoptotic cells were quantified using the Annexin V–FITC Apoptosis Detection Kit (A211, Vazyme) according to the manufacturer’s protocol. All experiments were performed in triplicate. Data were acquired and analyzed using Spectral Cell Analyzer SP6800Z (Sony) and analyzed by using the FlowJo V9 software.

### Analysis of lipid peroxidation.

Cells were washed once with PBS and incubated with PBS containing 5 μM C11-BODIPY (581/591) (D3861, Thermo Fisher Scientific) at 37°C for 30 minutes in the dark. Cells were then washed, harvested by trypsinization, washed twice with PBS, and resuspended in 500 to 1,000 μL fresh PBS. Lipid ROS levels were analyzed by CytoFLEX (Beckman Coulter) with FITC channel and Texas red channel.

### GSH assay.

GSH levels were measured using a GSH-Glo Glutathione Assay kit (V6911, Promega). In brief, cells were seeded at 2,000 cells per well in 96-well white plates. The medium was removed 20 hours later and indicated drugs were added. At 48 hours later, the medium was removed, and then 100 μL 1× GSH-GLO Reagent was added to each well and incubated for 30 minutes at room temperature. Then, 100 μL reconstituted Luciferin Detection Reagent was added to each well, mixed gently, and shaken slightly at room temperature for 15 minutes. Luminescence was detected by a multifunctional plate reader and normalized by cell viability respectively.

### TEM.

For ultrastructural analysis of mitochondria, TEM was used to observe ultrastructural of mitochondria. A2780 and A2780R cells were treated with or without trametinib for 48 hours, then harvested to be fixed with 2.5% glutaraldehyde in 0.1 M PBS (pH 7.4) at 4°C overnight. Then the samples were washed thrice with 0.1 M PBS and fixed with 1% OsO_4_ for 2 hours at 4°C. The samples were then dehydrated through an ethanol gradient and subsequently embedded in Spurr’s resin. Ultrathin sections were then collected and stained with either uranylacetate or lead citrate and examined using a transmission electron microscope (HT7800 120 kV, HITACHI).

### Immunoblot analysis and antibodies.

Briefly, cells were harvested and washed with PBS twice. Then cells were lysed using RIPA buffer. Protein concentrations were measured by Bradford assay (5000205, Bio-Rad). An equal amount of protein was subjected to SDS-PAGE with proper concentration and subsequently transferred to the PVDF membrane (Bio-Rad). After blocking in 5% BSA (A1933, MilliporeSigma) or 5% milk (9999, Cell Signaling Technology [CST]) for 3 hours and incubation with primary antibodies and secondary antibodies with appropriate concentration, immunoblotting was observed with ECL Western Blotting Detection Reagents (RPN2209, GE Healthcare Life Sciences, now Cytiva) in a Bio-Rad ChemiDoc MP imaging system. The primary antibodies used were as follows: SLC7A11 (12691S, 1:2,000), p-AKT (Ser473) (4060S, 1:1,000), AKT (4691S, 1:2,000), p-mTOR (5536S, 1:1,000), mTOR (2983S, 1:1,000), p-ERK1/2 (4370S, 1:2,000), ERK1/2 (4696S, 1:2,000), p-MEK (9154S, 1:1,000), MEK (9126S, 1:1,000), β-actin (3700S, 1:2,000), p-P70S6K (Thr389) (9205S, 1:1,000), P70S6K (9202S, 1:1,000), p-S6 (Ser235/236) (4858S, 1:1,000), S6 (2317S, 1:1,000), p-4EBP1 (Ser65) (9451S, 1:1,000), and 4EBP1 (9644S, 1:1,000) were obtained from CST. GPX4 (ab125066, 1:2,000) and the anti-rabbit secondary antibody (ab205718, 1:20,000) were purchased from Abcam. The anti-mouse secondary antibody (NA931, 1:5,000) was purchased from GE Healthcare Life Sciences.

### qRT-PCR.

Total RNA was extracted using the RNeasy Mini Kit (74106, QIAGEN); cDNA was subsequently produced using TranScript All-in-One First-Strand cDNA Synthesis SuperMix for RT-PCR (One-Step gDNA Removal) (TransGen Biotech). qRT-PCR was conducted following the instructions of PerfectStart Green qPCR SuperMix (TransGen Biotech). 18S was used as an endogenous housekeeping gene for normalization. The primer pairs of the genes used for quantitative qRT-PCR are as follows: SLC7A11 5′-ATGCAGTGGCAGTGACCTTT-3′ and 5′-GGCAACAAAGATCGGAACTG-3′; 18S 5′-GTAACCCGTTGAACCCCATT-3′and 5′-CCATCCAATCGGTAGTAGCG-3′; Firefly-luc 5′-GGTACTGTTGGTAAAGCCAC-3′ and 5′-CTCTTCATAGCCTTATGCAG-3′; Renilla-luc 5′-CACTGGGCAGGTGTCCACTC-3′ and 5′-GTTCTGGATCATAAACTTTC-3′. The mRNA levels of these genes were determined as the mean of the Ct values obtained from the couple of primers. Data are described as relative mRNA expression levels.

### Plasmid construction and virus infection.

SLC7A11-knockdown cell lines were generated using CRISPR/Cas9 technology. For SLC7A11 gene knockdown, single-guide RNA (sgRNA) sequences were designed using the Optimized CRISPR Design (http://chopchop.cbu ib.no/) and inserted into the lentiCRISPR v2 vector (Addgene plasmid 52961) containing the *Streptococcus pyogenes* Cas9 nuclease gene. gRNA sequences targeting the SLC7A11 are as follows: SLC7A11 sgRNA#1: 5′-CACCGACCATAGTAGGGACACACGG-3′ and 5′-AAACCCGTGTGTCCCTACTATGGTC-3′; SLC7A11 sgRNA#2: 5′-CACCGTATGGGACAAGAAACCCAGG-3′ and 5′-AAACCCTGGGTTTCTTGTCCCATAC-3′. ShRNA sequences targeting human 4EBP1 (TRCN 0000040203, MilliporeSigma) were inserted into PLKO.1 plasmid (Addgene plasmid 10878), and the knockdown effect of shRNA sequences targeting human 4EBP1 has been validated in previous studies (49, 50). The plasmids of pCDH-EF1-Neo-SLC7A11-myc and pCDH-EF1-Neo were gifts from Zhu Xiaofeng, from Sun Yat-sen University Cancer Center in Guangzhou in China. Human 4EBP1 was amplified from HEK293T cDNA and then inserted into the pCDH-CMV-MCS-EF1-copGFP-T2A-Puro lentiviral expression vector (System Biosciences) to obtain pCDH-4EBP1 plasmid. Then we generated the mutant of pCDH-4EBP1 by using the KOD -Plus- Mutagenesis Kit (SMK-101, TOYOBO) to obtain pCDH-4EBP1-4A plasmid (containing 4 mutation sites, including T37A, T46A, S65A, T70A).

The lentiviral vectors were transfected into HEK293T packaging cells with Lipofectamine 2000 (11668019, Thermo Fisher Scientific). The viral supernatants were passed through a 0.45 μm nitrocellulose filter (MilliporeSigma) and were used to infect target cells. After transfected for 48 hours, stably transfected cells were selected with 1.0 μg/mL puromycin (MilliporeSigma) for 4 days or 1 mg/mL G-418 disulfate (T6512, Target Mol) for 1 week.

### Luciferase reporter assay.

The plasmids of SLC7A11-FL, SLC7A11-T1, SLC7A11-T2, and SLC7A11-T3 are gifts from Wang Yang at Dalian Medical University in Dalian in China and have been described in a previous study (20). Luciferase reporter assay was performed using a Dual-Luciferase Reporter Assay System (E1910, Promega) according to the product instructions. Briefly, 3 × 10^5^ targeted cells seeded in 12-well plates were transfected with 1 μg targeted plasmids and 100 ng pRL-TK for 48 hours. The cells were washed once with PBS, and 100 μL lysis buffer each well was added to lyse cells for 1 hour at room temperature. Then we extracted 20 μL supernatant and split into 96-well white plates for subsequent luciferase activity measurement, following product instructions. Luminescence from 3 independent samples was recorded using a multifunctional plate reader.

### Mouse xenograft experiment.

The 6-week-old female nude mice used in this study were purchased from Beijing Vital River Laboratory Animal Technology Co. Tumor size and body weight were measured twice to 3 times a week, and volume of tumor was calculated with the formula: width^2^ × length × 0.537, where length represents the longest diameter and width means the shortest diameter.

For the SKOV3 tumor xenograft experiment, 4 × 10^6^ SKOV3 cells were injected subcutaneously in the right flank of the BALB/c nude mice. For PDX mouse models, PDX-POVC15 tumor masses were performed to passage into NOD/SCID mice (Beijing Vital River Laboratory Animal Technology Co.). When the tumors reached approximately 100 mm^3^, the mice were randomly divided into 4 groups for treatment: (a) vehicle, (b) trametinib, (c) MK2206, and (d) combination (trametinib and MK2206). Trametinib was dissolved in 0.5% methylcellulose and 0.2% Tween-80, and MK2206 was prepared in PBS containing 30% captisol. Drug dosages were given as follows: trametinib, 0.25 mg/kg every other day (intraperitoneal injection); MK2206, 60 mg/kg in SKOV3 and 90 mg/kg in PDX-POVC15 every other day (orally). When tumor volume of the vehicle group reached about 1,000 mm^3^, mice were sacrificed using CO_2_, and tumors were collected for further analysis.

For animal survival study, PDX-POVC15 tumor masses were performed to passage into NOD/SCID mice. When the tumors reached approximately 100 mm^3^, the mice were randomly divided into 4 groups to receive therapy as described in xenograft experiments. Drug treatment was withdrawn until the tumor volume of the first mouse reached 1,000 mm^3^. Animal survival of every mouse was evaluated from the first day of treatment until the tumor volume reached 1,000 mm^3^, followed by detection of the levels of ALT, AST, BUN, and creatinine in the serum of PDX-POVC15.

### IHC staining.

IHC staining was conducted using standard procedures. Xenograft tumors were harvested, fixed with 10% formalin immediately, and embedded in paraffin. After deparaffinization, rehydration, and antigen retrieval by heat-induced epitope retrieval, endogenous peroxidase was blocked with 3% H_2_O_2_ at room temperature. Antibodies specific to SLC7A11 (1:100; 12691S, CST), p-4EBP1 (1:800; 2855S, CST) and GPX4 (1:500; 52455S, CST) were used in this study, and tissues were incubated overnight at 4°C. On the second day, after incubation of secondary antibodies (Dako REAL HRP Rabbit detection kit) for 30 minutes, the DAB reagent kit (ZLI-9019, ZSGB-BIO) was used as chromogen, and hematoxylin (ZLI-9609, ZSGB-BIO) was used as counterstain. Histoscore was a multiplicative index of the intensity of staining and the proportion of positive tumor cells. The intensity was graded as follows: 0, negative staining; 1, mild staining; 2, moderate staining; 3, strong staining. The percentage of stained cells was defined as follows: 1, less than 10%; 2, 10%–50%; 3, 50%–75%; 4, more than 75%.

For human OV tissue analysis, the optimal cutoff point of SLC7A11 expression was performed based on X-tile software (X-tile 3.6.1) (51), which was used to classify tumors into high-expression group and low-expression group. In this study, SLC7A11 histoscore with 0~3 was identified as SLC7A11-low expression (24 cases), while histoscore with 4~12 was considered as SLC7A11 high expression (20 cases). GPX4 histoscore with 0~4 was identified as GPX4-low expression (17 cases) while histoscore with 5~12 was considered as GPX4-high expression (27 cases).

### Statistics.

Comparisons between 2 groups were analyzed by 2-tailed Student’s *t* tests with GraphPad Prism 9.0 Software. Comparisons among more than 2 groups were analyzed using 1-way ANOVA. Survival curves were described by Kaplan-Meier plots and compared with the log-rank test. Two-way ANOVA was used to calculate differences between 2 curves with multiple time or concentration points. Data are presented as mean ± SD unless otherwise stated, with at least 3 biological replicates in each group. *P* values less than 0.05 were considered statistically significant.

### Study approval.

All procedures of animal work were performed in compliance with standard procedures approved by the Institutional Animal Care and Use Committee of Sun Yat-sen University. We have obtained all human material under approval by the medical ethics committee of the Sun Yat-sen University Cancer Center and with written patient informed consent.

### Data availability.

The key raw data are available at Research Data Deposit public platform (https://www.researchdata.org.cn) with approval number RDDB2024608391. Values for all data points in graphs are reported in the supplemental Supporting Data Values file.

## Author contributions

JY and JT designed and conceived the study. JY conducted the most experiments and prepared the manuscript. JT supervised the project. JT, YX, and JC revised the manuscript. YH, XZ, RX, and JC contributed to the technical support and animal work. PD, SL,YS, PW, YW, CT, and JC provided material support. JHH, JYC, PG, KXYC, BTT, QY, YX, XX, and CKO provided a critical reading of the manuscript. All the authors have given their consent to publish this study.

## Supplementary Material

Supplemental data

Unedited blot and gel images

Supplemental table 1

Supporting data values

## Version 1 06/06/2024

In-Press Preview

## Version 2 07/22/2024

Electronic publication
