# Supplementary material for: 4EBP1-mediated SLC7A11 protein synthesis restrains ferroptosis triggered by MEK inhibitors in advanced ovarian cancer
Source: JCI Insight. 2024 Jun 6;9(14):e177857. doi: 10.1172/jci.insight.177857 (PMC11383183; doi:10.1172/jci.insight.177857)
Supplement: Supplemental data [file jciinsight-9-177857-s047.pdf]

Supplementary Figure 1

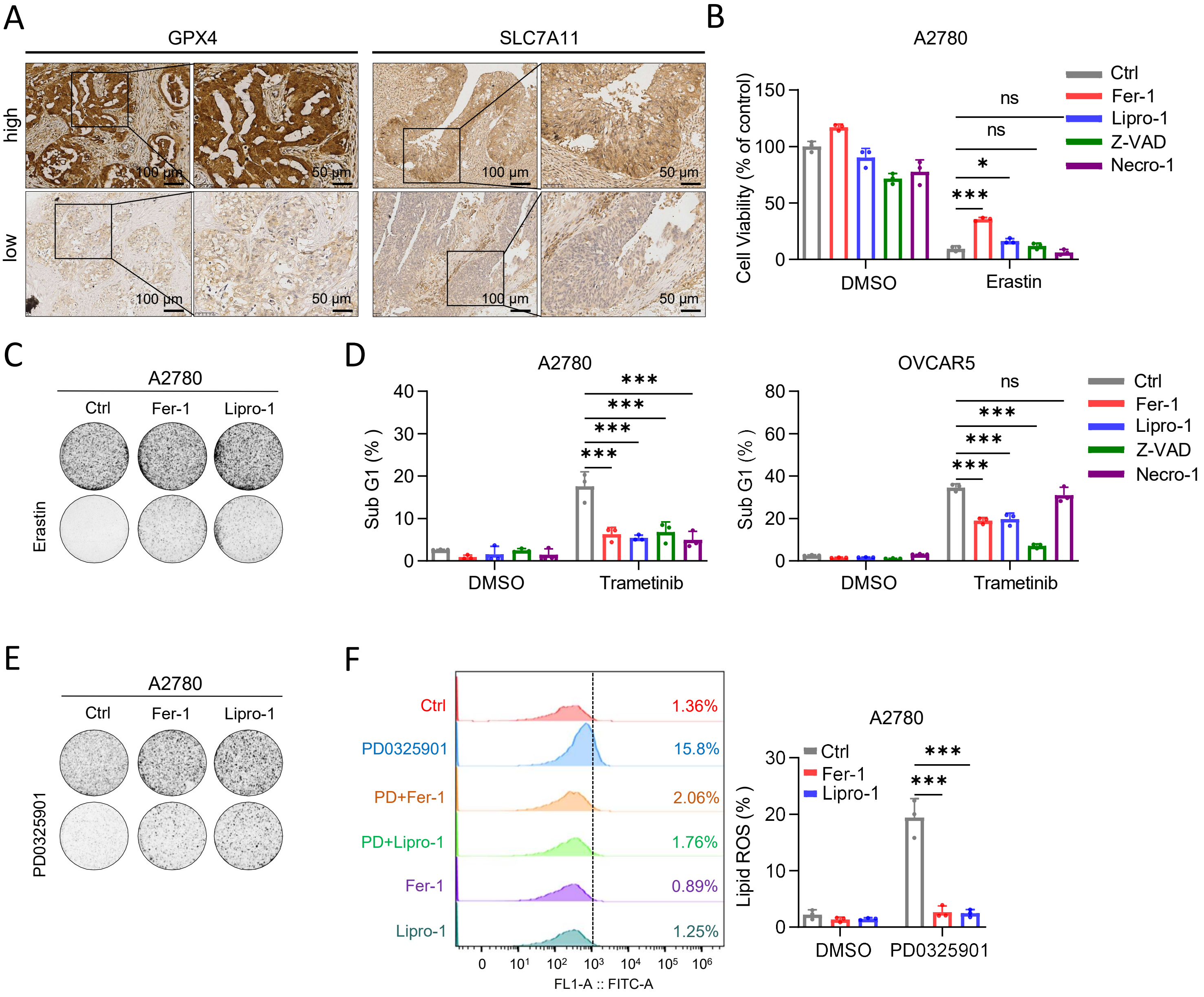

Supplementary Figure 1. MEK inhibitors trigger ferroptosis in OV. (A) Representative IHC of OV patient tissues that high and low expressed GPX4 or SLC7A11 in figure 1C-1D. Scale bar: 100 μm (left) and 50 μm (right). **(B)** Cell viability assay of A2780 cells treated with vehicle or the erastin (500 nM) in the absence or presence of Fer-1 (2μM), Lipro-1 (100nM), Necro-1 (5μM) and Z-VAD-FMK (5μM) for 72hours. **(C)** Colony formation assay of A2780 cells treated with vehicle or the erastin in the absence or presence of Fer-1 or Lipro-1. **(D)** Sub-G1 population analysis in A2780 and OVCAR5 cells treated with vehicle or the Trametinib(200nM in A2780 and 500nM in OVCAR5) in the absence or presence of Fer-1 (2μM), , Lipro-1 (100nM), Necro-1 (5μM) and Z-VAD-FMK (5μM) for 72 hours. Data are shown as mean ± SD (n=3). (E) Colony formation assay of A2780 cells treated with vehicle or the PD0325901 (100 nM) in the absence or presence of Fer-1 (2μM) or Lipro-1 (100nM). (**F**) Detection of lipid ROS level in A2780 treated with PD0325901 (100 nM) in the absence or presence of Fer-1 (2μM) or Lipro-1 (100nM). Data were shown as mean ± SD (n=3). (B, D and F) *P* values were determined by 1-way ANOVA with Bonferroni's post hoc test. ns, not significant, **P* < 0.05, ****P* < 0.001.

Supplementary Figure 2

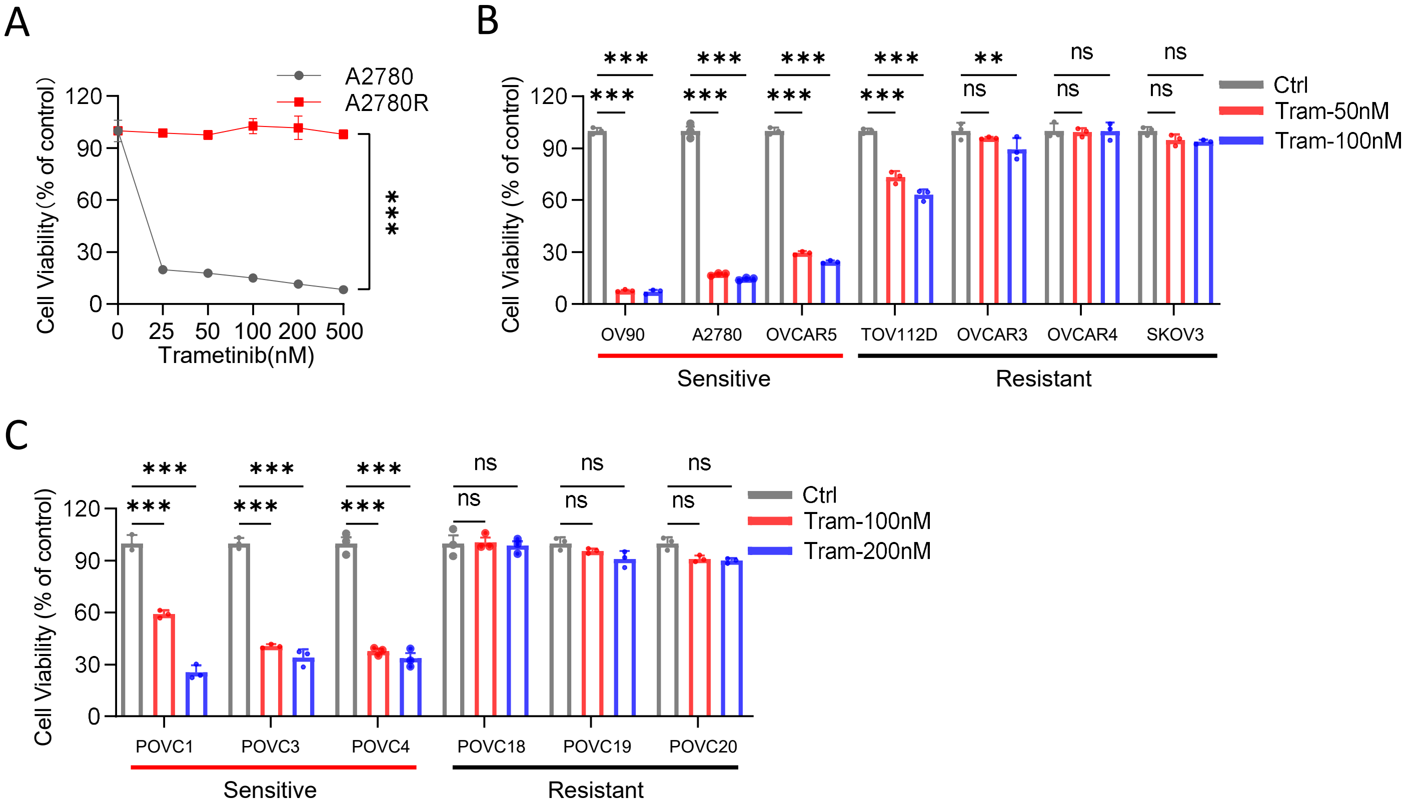

Supplementary Figure 2. The sensitivity to trametinib of OV cells. (A) Cell viability assay of A2780 and A2780R treated with vehicle or different dosages of trametinib for 96 hours. **(B)** Cell viability assay of 7 commercial OV cell lines treated with vehicle or trametinib. **(C)** Cell viability assay of 6 patient-derived primary cells treated with vehicle or trametinib. Data were shown as mean ± SD (n=3). (A, B and C) *P* values were determined by 1-way ANOVA with Bonferroni's post hoc test. ns, not significant, **P* < 0.05, ****P* < 0.001.

Supplementary Figure 3

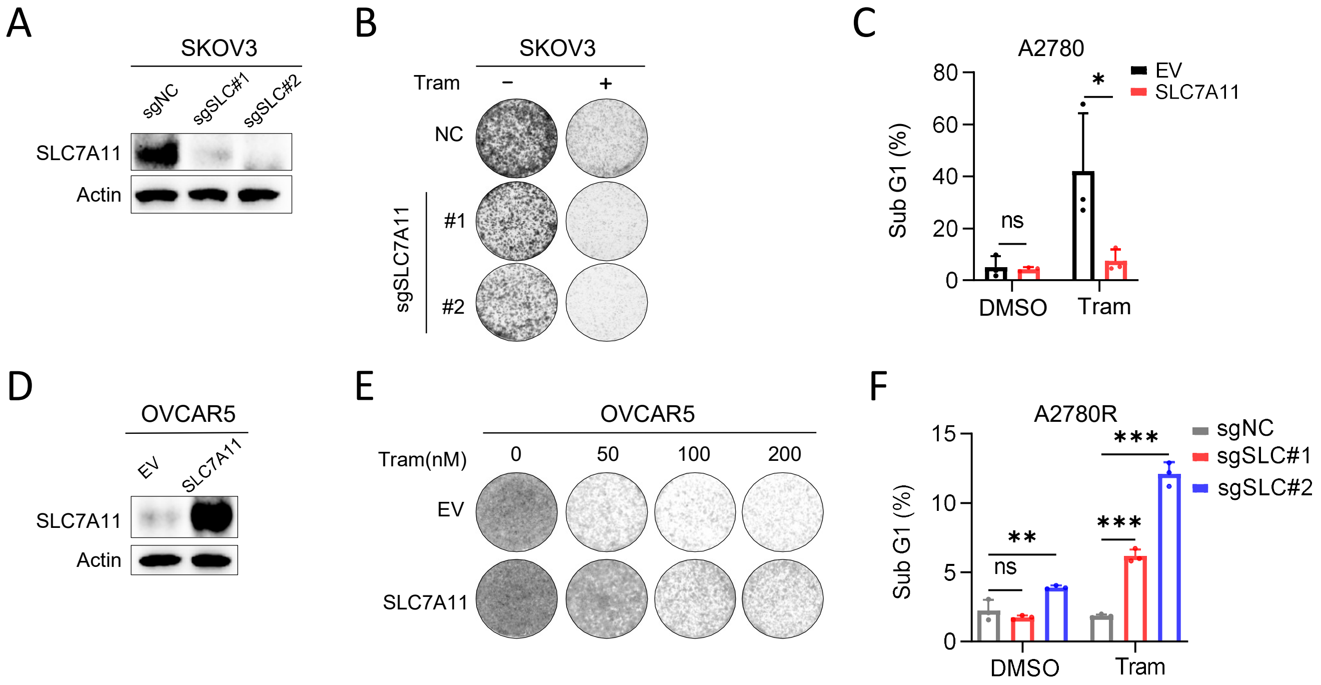

Supplementary Figure 3. SLC7A11 protein synthesis dictates the sensitivity of OV cells to ferroptosis triggered by MEK inhibitors. (A) The effect of CRISPR/Cas9-mediated SLC7A11 knockdown (sgSLC#1 and sgSLC#2) evaluated by immunoblot analysis in SKOV3 **(B)** Colony formation assay of the effect of SLC7A11 ablation and control (sgNC) on trametinib sensitivity in SKOV3 cells. The concentration of trametinib is 2.5μM. **(C)** Sub-G1 population analysis in A2780 cells with empty vector(EV) or SLC7A11 overexpression(SLC7A11) under trametinib treatment (100nM). Data are shown as mean ± SD (n=3). **(D)** The effect of SLC7A11 overexpression evaluated by immunoblot analysis in A2780. **(E)** Colony formation analysis in A2780 cells with EV or SLC7A11 overexpression under trametinib treatment at indicated concentrations. **(F)** Sub-G1 population analysis in A2780R cells with sgNC or SLC7A11 ablation under trametinib treatment (10μM). Data are shown as mean ± SD (n=3). (C) P-values were determined by unpaired Student's t test. (F) P values were determined by 1-way ANOVA with Bonferroni's post hoc test. **P* < 0.05, ***P* < 0.01, ****P* < 0.001.

Supplementary Figure 4

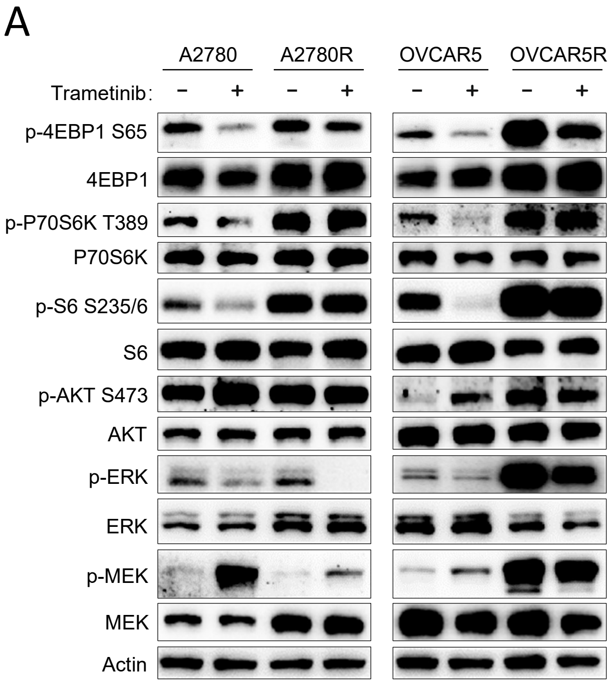

Supplementary Figure 4. mTOR-4EBP1 pathway modulates SLC7A11 protein synthesis to promote ferroptosis escape upon trametinib treatment. (A) Immunoblot analysis of 4EBP1, S6K, S6, AKT, ERK and MEK activity in A2780, A2780R, OVCAR5 and OVCAR5R cells treated with vehicle or 200nM trametinib.

Supplementary Figure 5

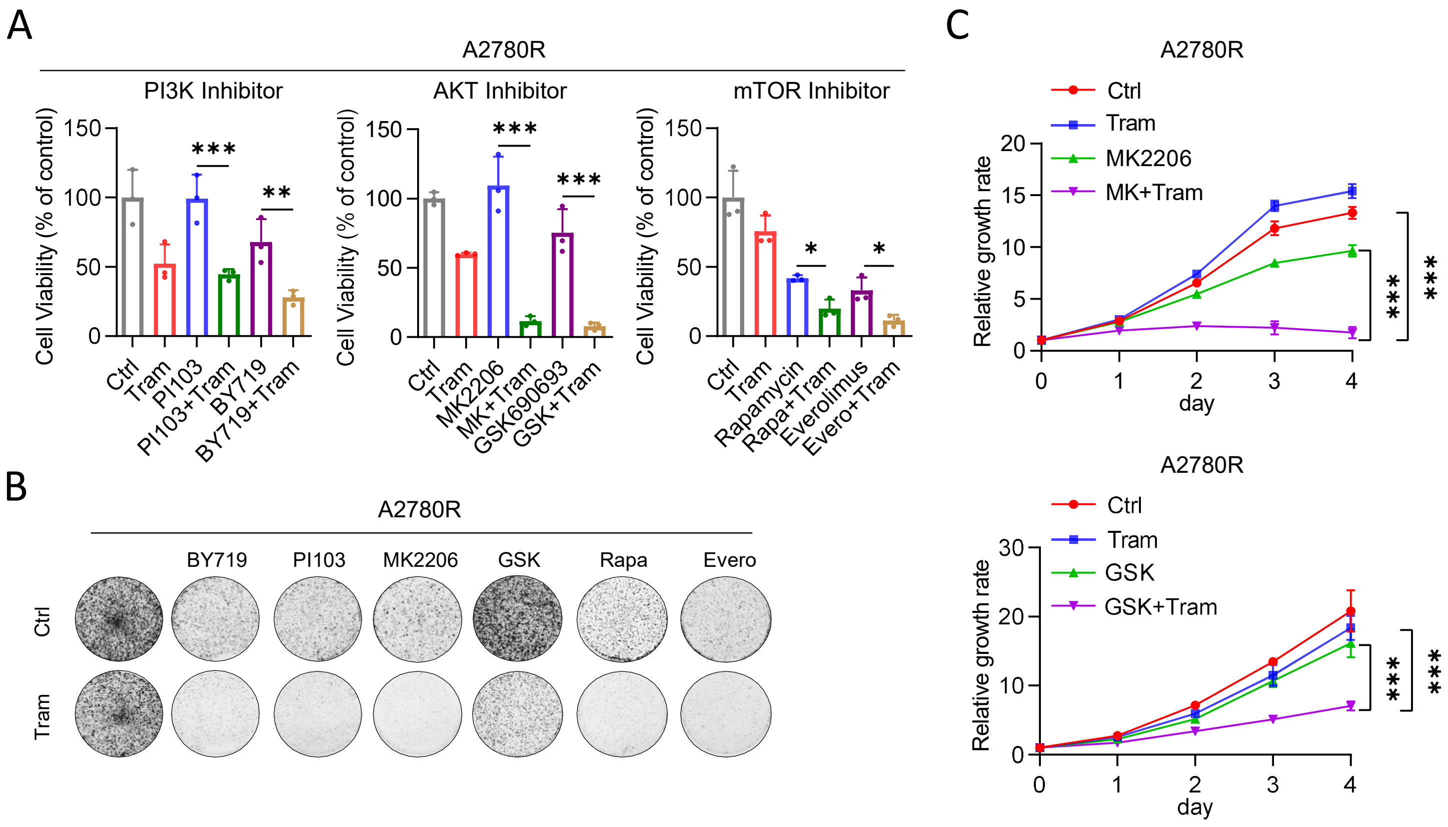

Supplementary Figure 5. Targeting PI3K/mTOR signaling sensitized resistant cells to ferroptosis induced by MEK inhibitors. (A-B) Cell viability (A) and colony formation analysis (B) of A2780R treated with 500nM trametinib with or without PI3K/AKT/mTOR inhibitors. BY719 (5 μM), PI103 (2 μM), MK2206 (5μM), GSK690693 (GSK) (10 μM), Rapamycin (Rapa) (1 μM), Everolimus (Evero) (1 μM). **(C)** Growth curves of A2780R treated with either vehicle, trametinib, AKT inhibitors (MK2206, 5 μM and GSK690693, 10 μM) or their combination. Data were shown as mean ± SD (n=3). (A) *P* values were determined by unpaired Student's t test. (C) *P* values were determined by 2-way ANOVA with Tukey's post hoc test. *P < 0.05, **P < 0.01, ***P < 0.001.

Supplementary Figure 6

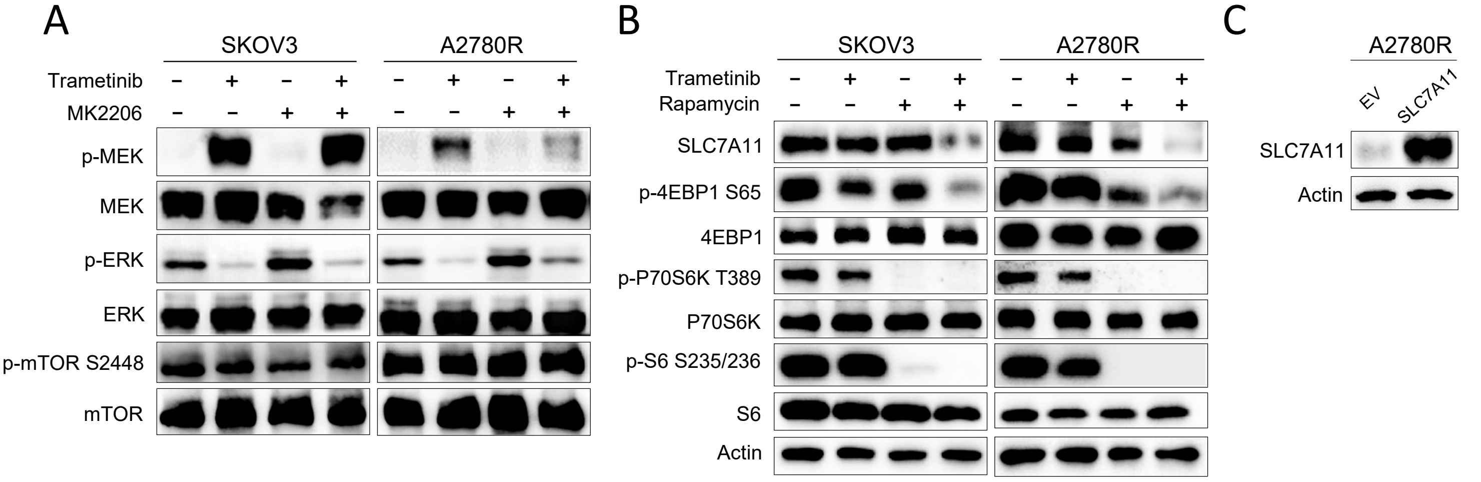

Supplementary Figure 6. Co-targeting AKT and MEK suppresses the protein synthesis of SLC7A11 via inhibition of mTOR-4EBP1 activity. (A) Immunoblot analysis of the activity of MEK, ERK and mTOR in SKOV3 and A2780R cells under the treatment of trametinib (500nM) and MK2006 (5μM). **(B)** Immunoblot analysis of SLC7A11 as well as the activity of 4EBP1, P70S6K and S6 in SKOV3 and A2780R cells under the treatment of trametinib (500nM) and Rapamycin (2.5μM in SKOV3 and 5μM in A2780R). **(C)** The effect of SLC7A11 overexpression evaluated by immunoblot analysis in A2780R.

Supplementary Figure 7

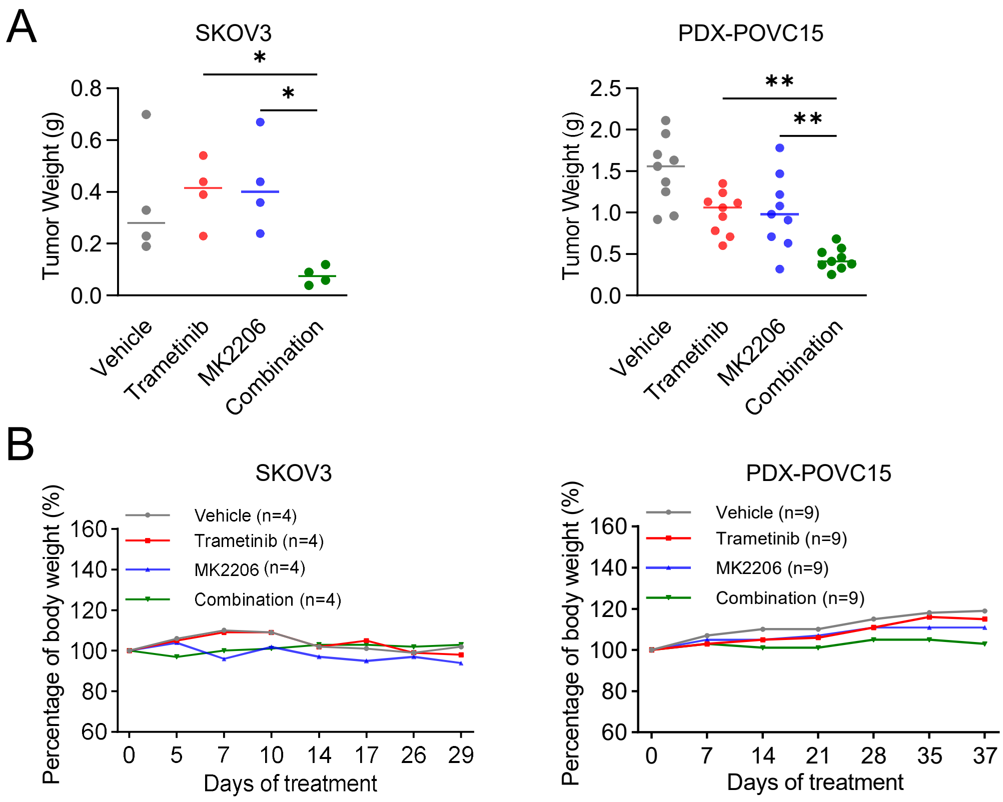

Supplementary Figure 7. AKT inhibitor sensitizes OV to MEK inhibitor-mediated ferroptosis *in vivo*. (A) The weights of the harvested SKOV3 and PDX-POVC15 tumors at the termination day. Data are presented as mean ± S.D. *$P < 0.05$, **$P < 0.01$ by 1-way ANOVA with Bonferroni's post hoc test. **(B)** The change of body weight of SKOV3 xenografts and PDX-POVC15 from the experiment described in Figure 6A-B respectively.
