# Supplementary material for: 4EBP1-mediated SLC7A11 protein synthesis restrains ferroptosis triggered by MEK inhibitors in advanced ovarian cancer
Source: JCI Insight. 2024 Jun 6;9(14):e177857. doi: 10.1172/jci.insight.177857 (PMC11383183; doi:10.1172/jci.insight.177857)

Unedited blot and gel images

Full unedited gel for **Figure 3A**

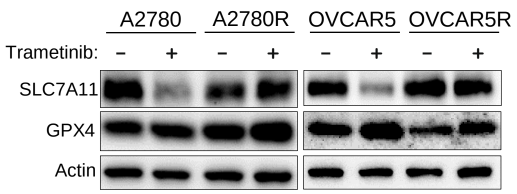

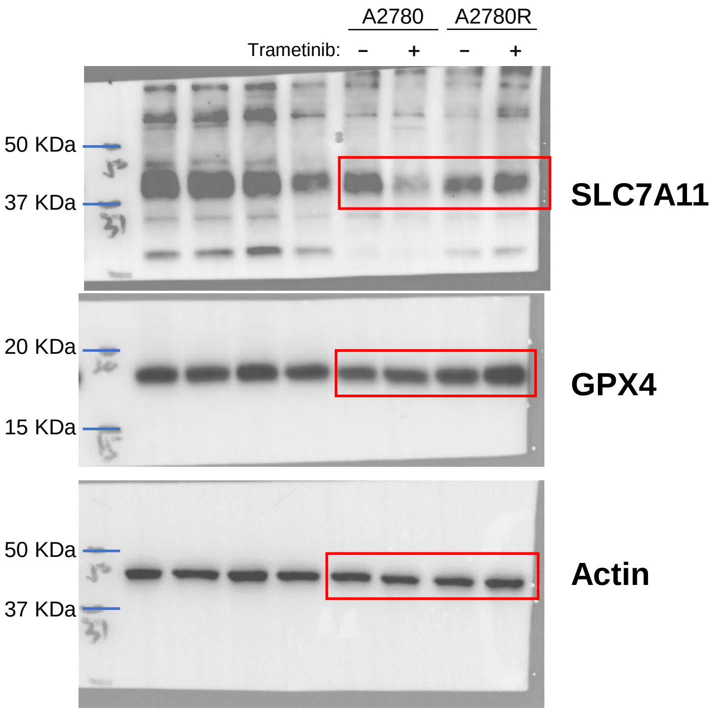

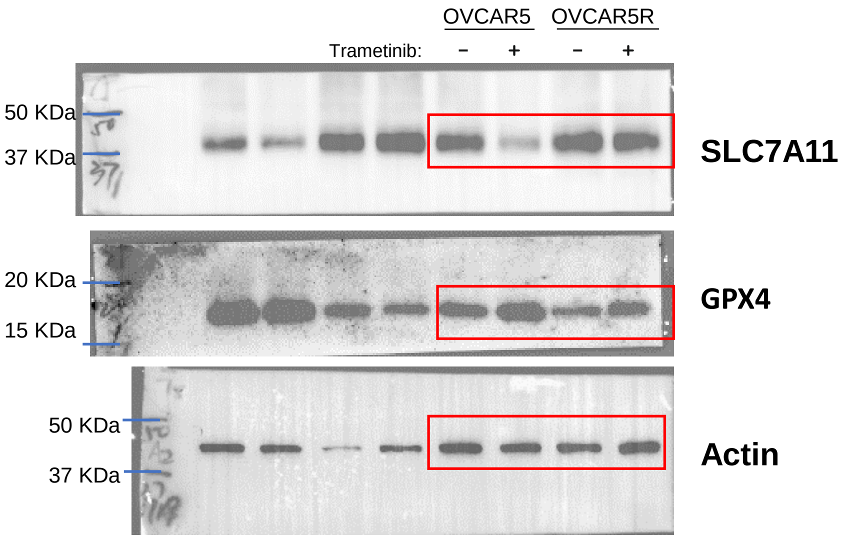

Full unedited gel for **Figure 3C**

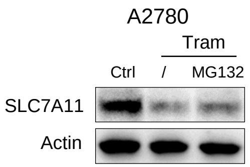

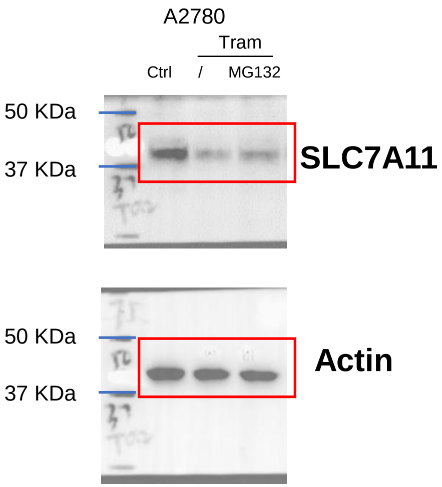

Full unedited gel for **Figure 3G**

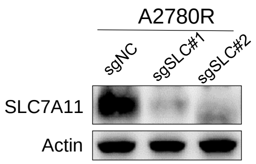

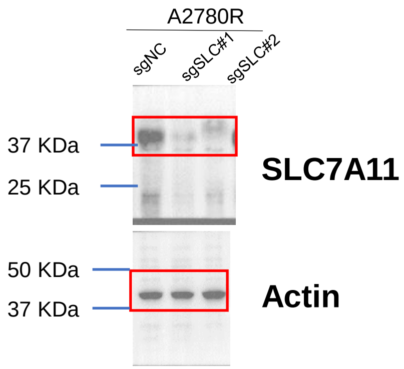

Full unedited gel for **Figure 3K**

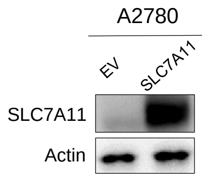

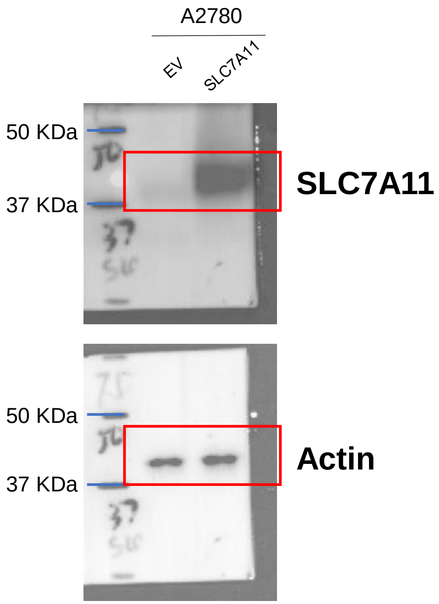

Full unedited gel for **Figure 4A**

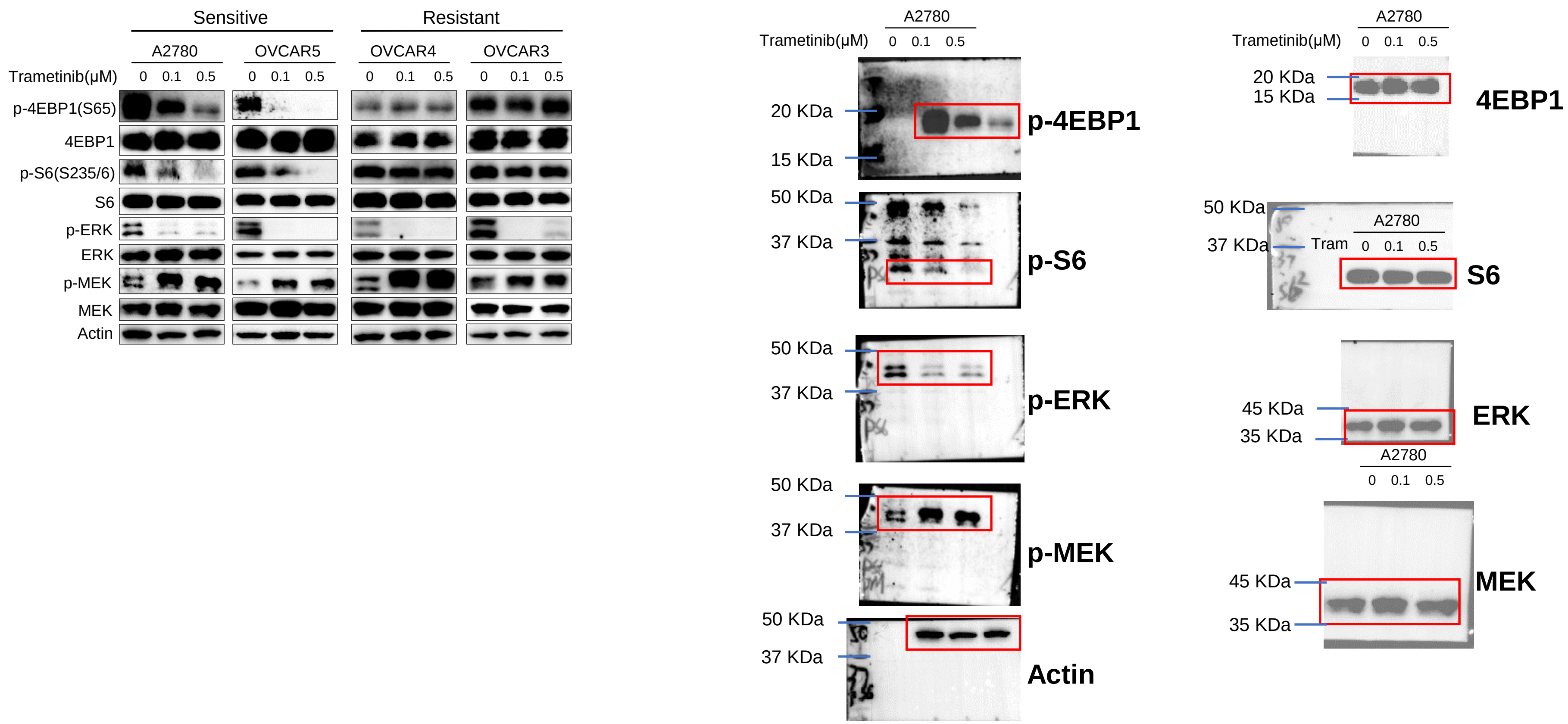

Full unedited gel for **Figure 4A**

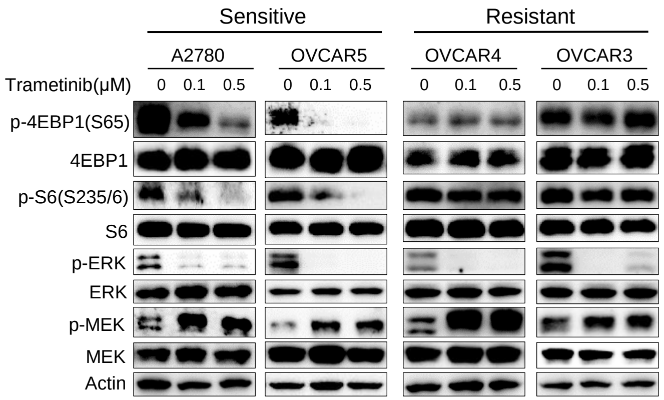

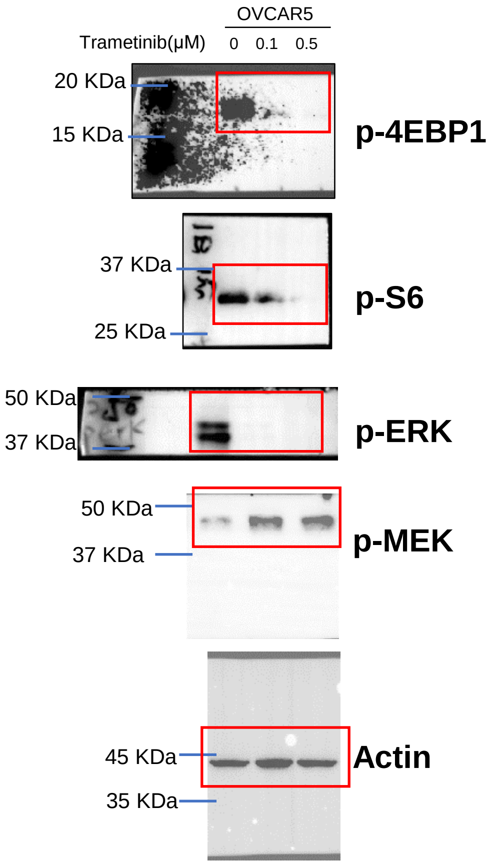

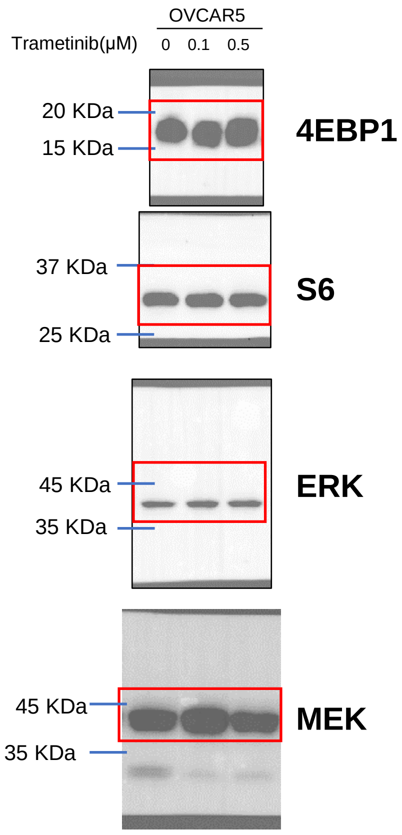

Full unedited gel for **Figure 4A**

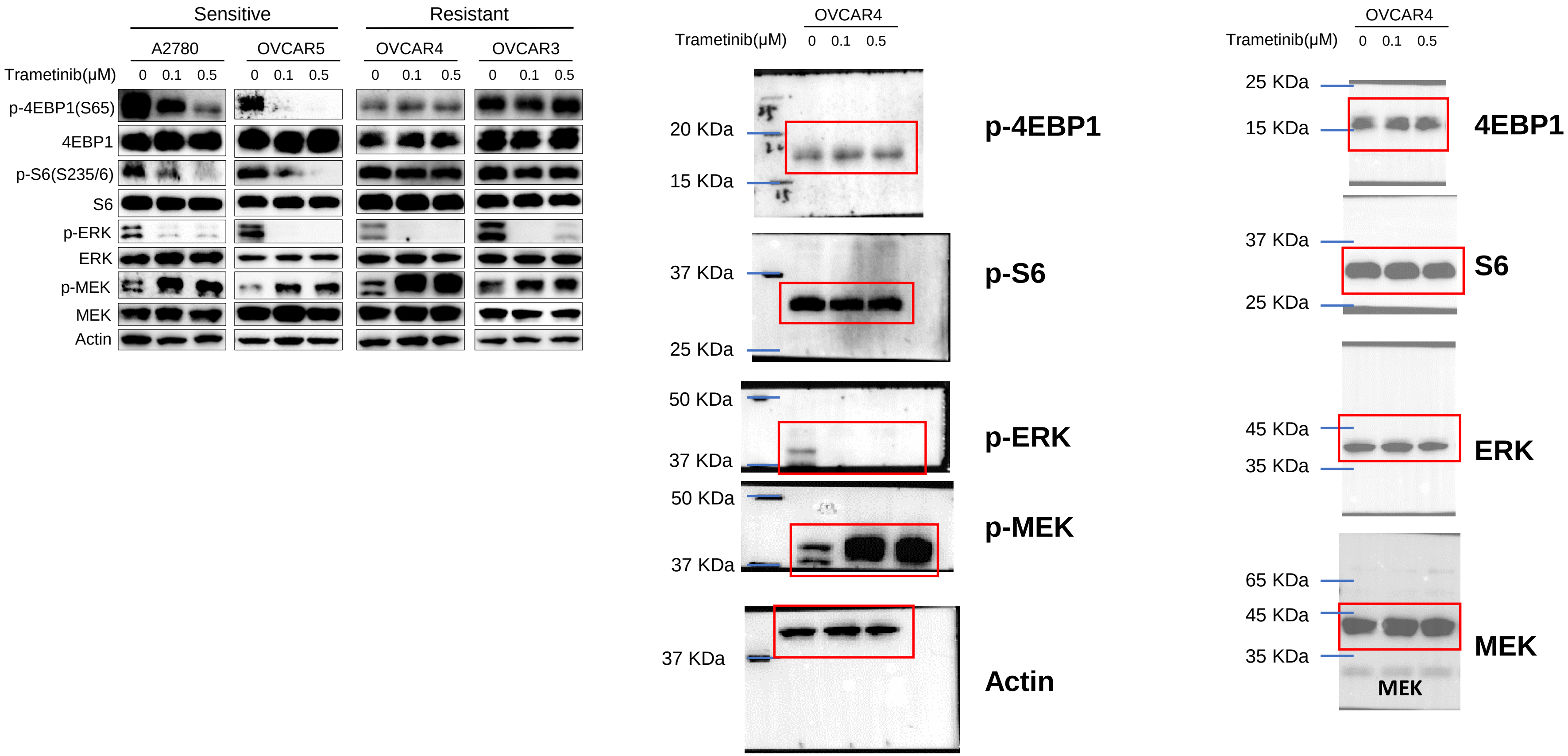

Full unedited gel for **Figure 4A**

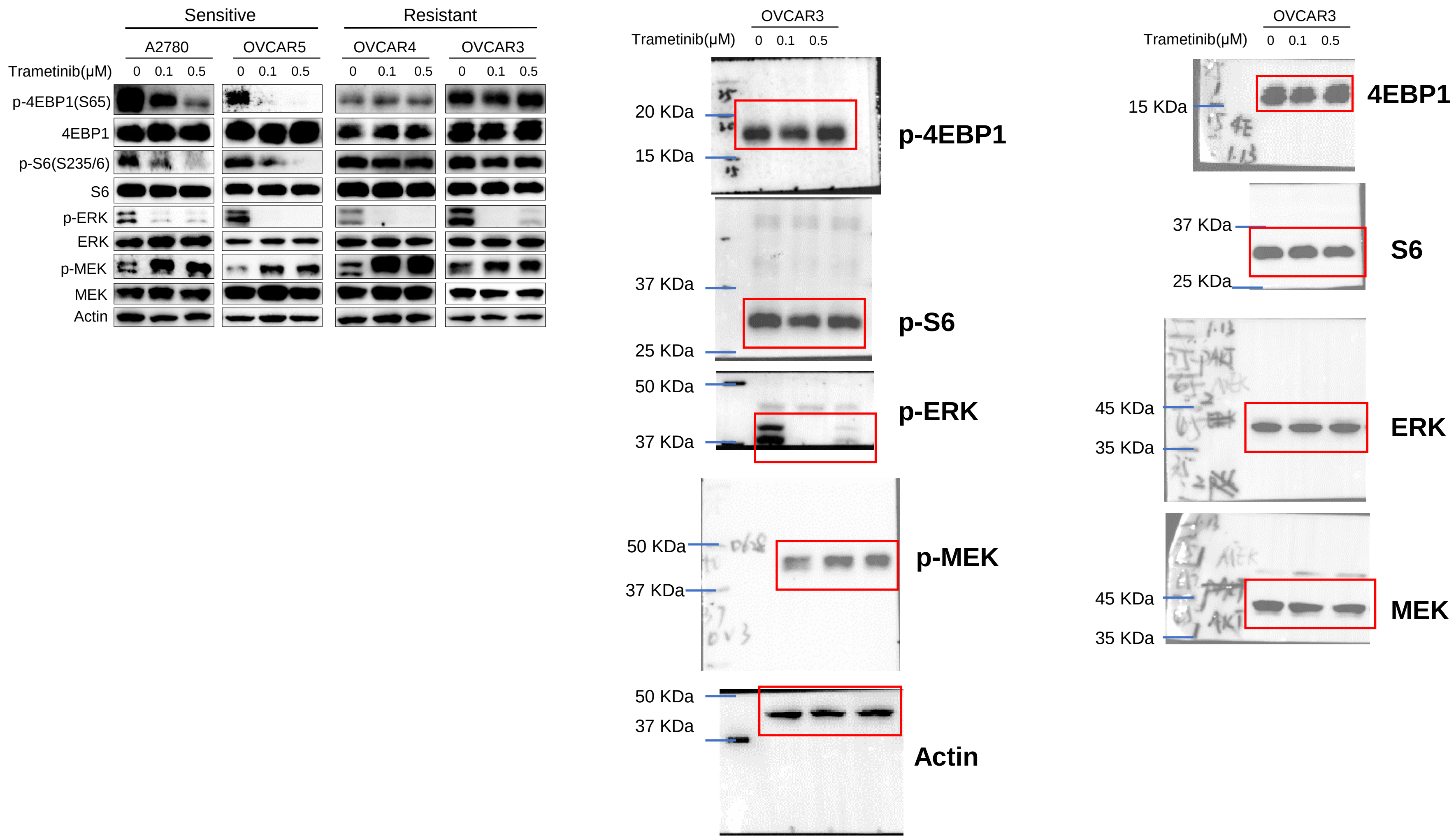

Full unedited gel for **Figure 4B**

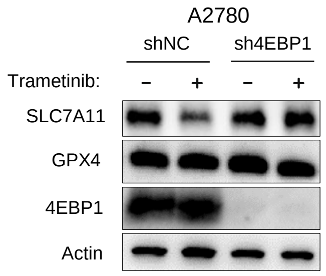

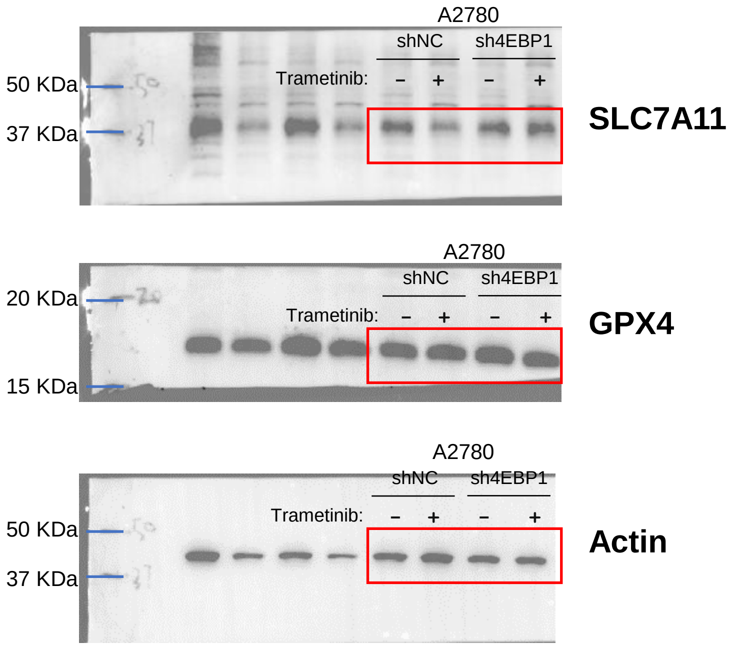

From the same gel

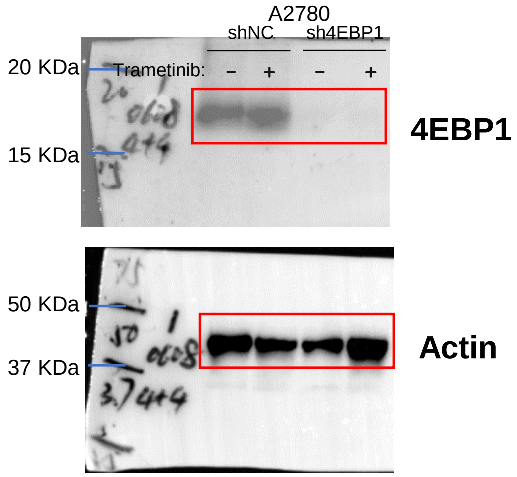

From the same gel

Full unedited gel for **Figure 4D**

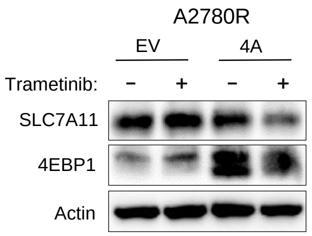

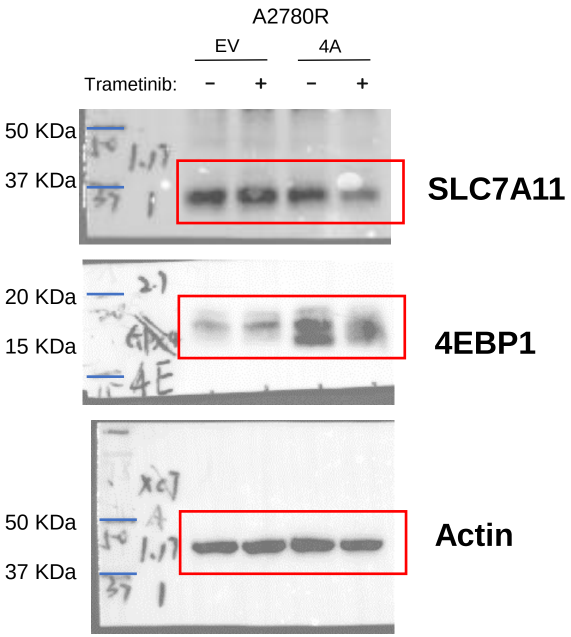

Full unedited gel for **Figure 6B**

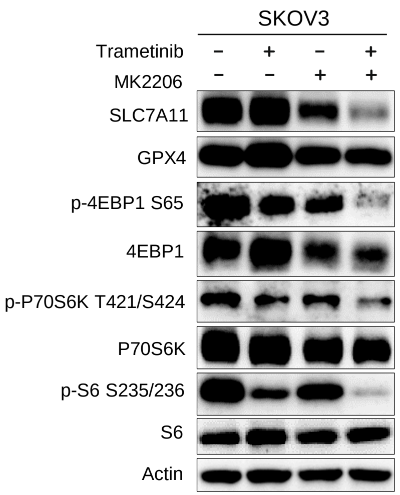

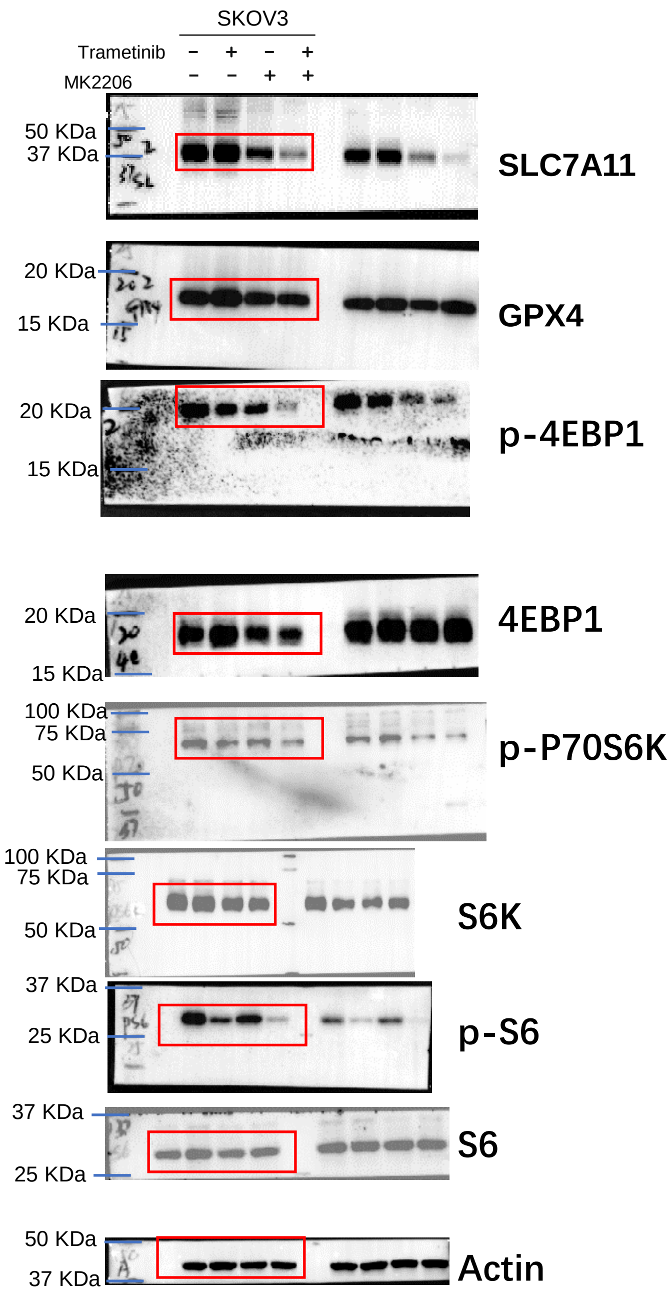

Full unedited gel for **Figure 6B**

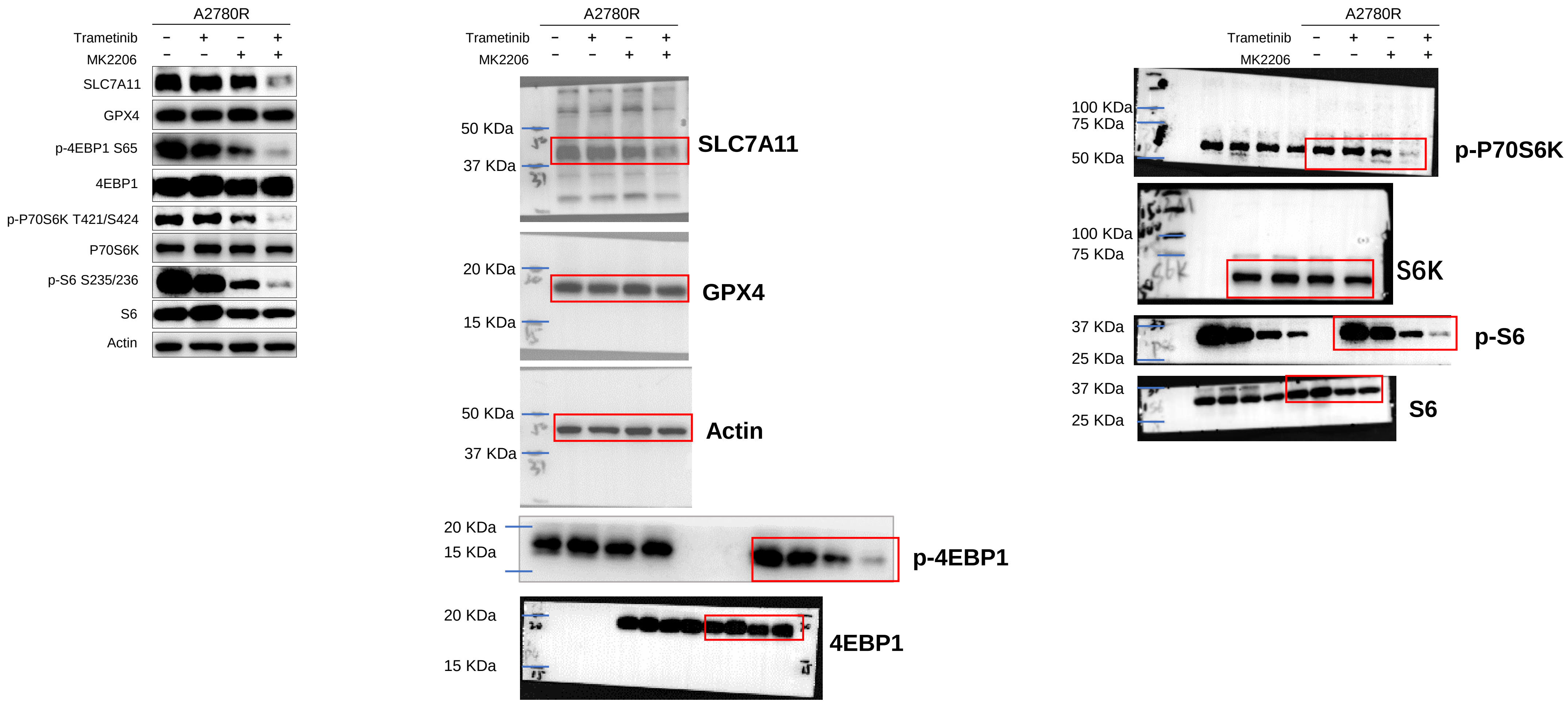

Full unedited gel for **Figure 6C**

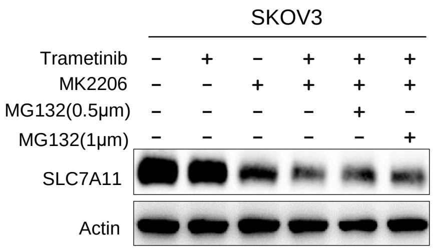

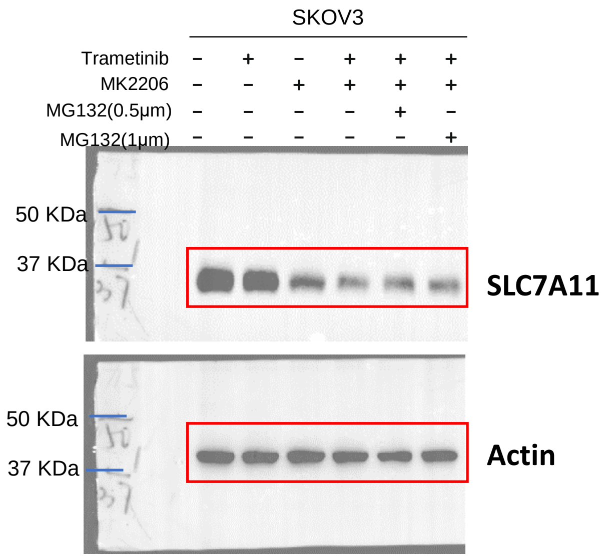

Full unedited gel for **Figure 6D**

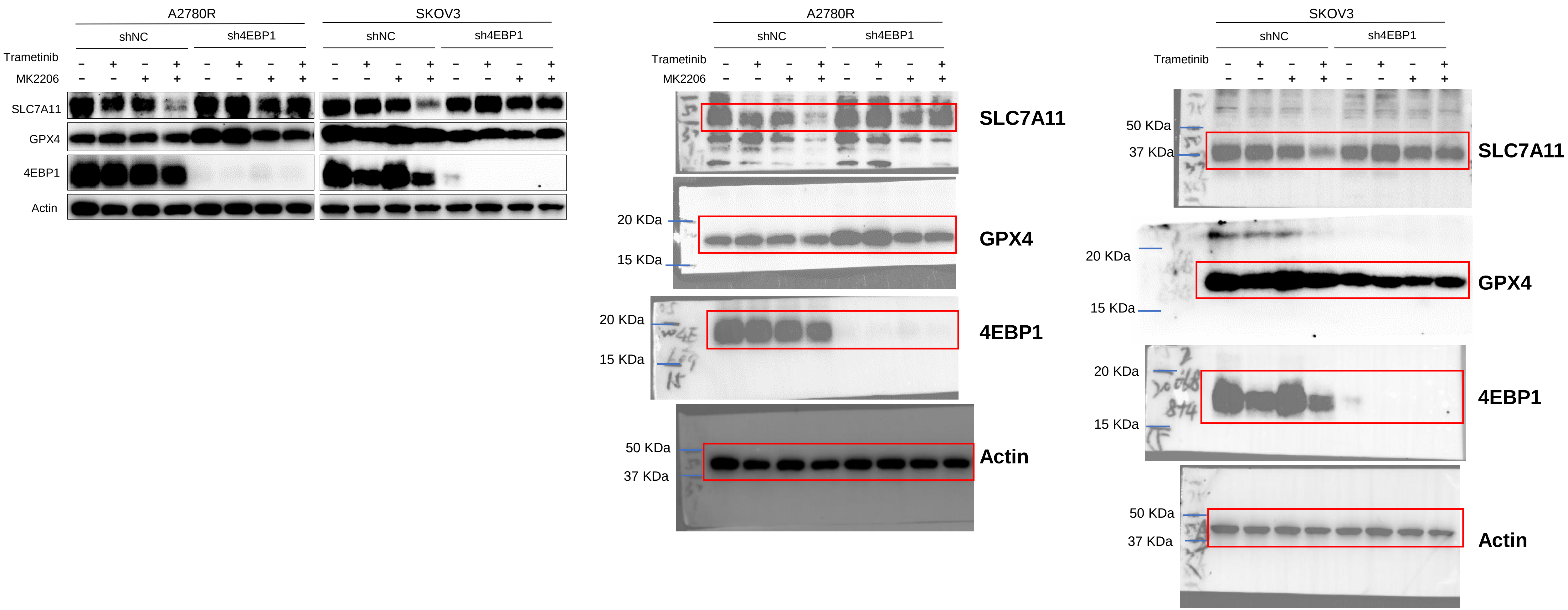

Full unedited gel for **Supplementary Figure 3A and 3D**

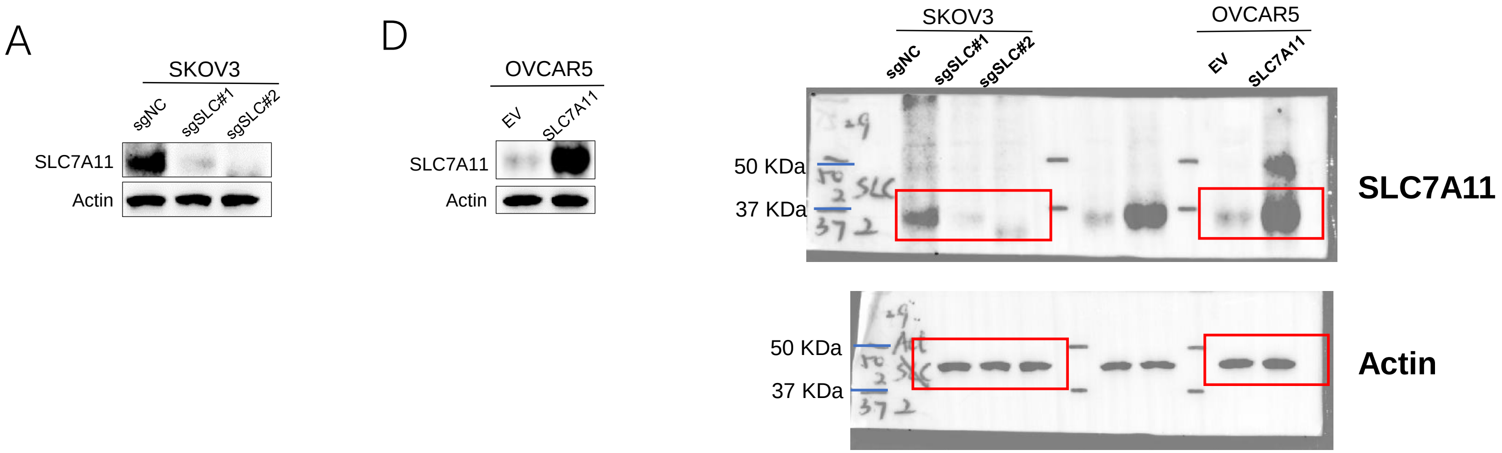

Full unedited gel for **Supplementary Figure 4A**

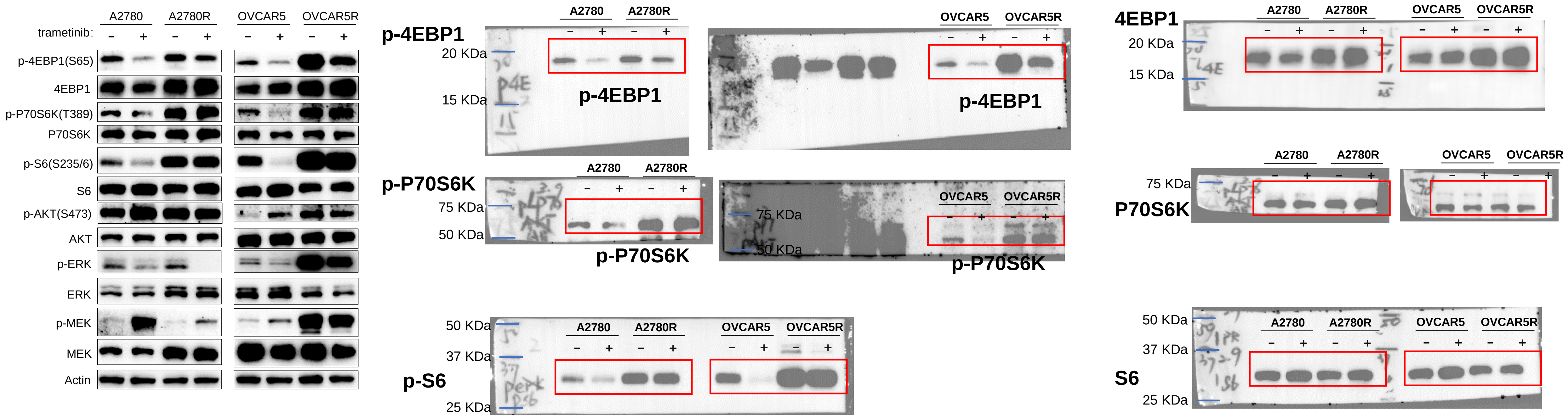

Full unedited gel for **Supplementary Figure 4A**

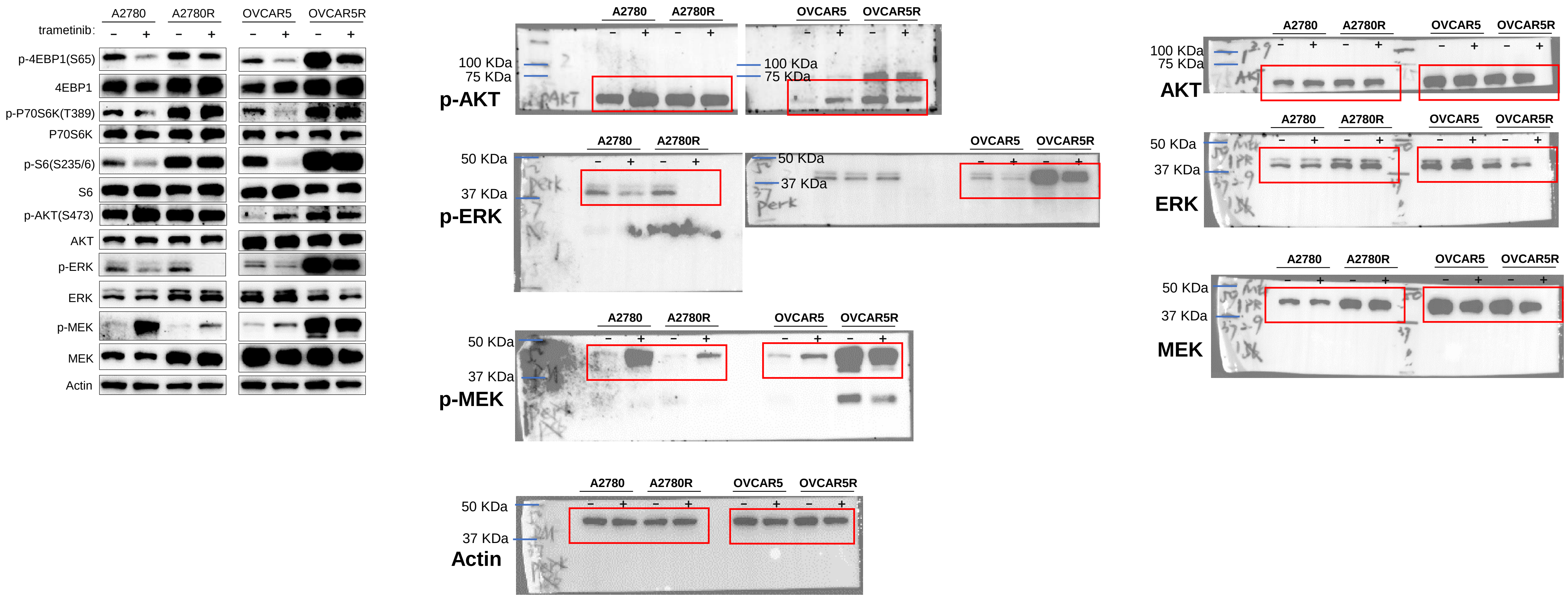

Full unedited gel for **Supplementary Figure 6A**

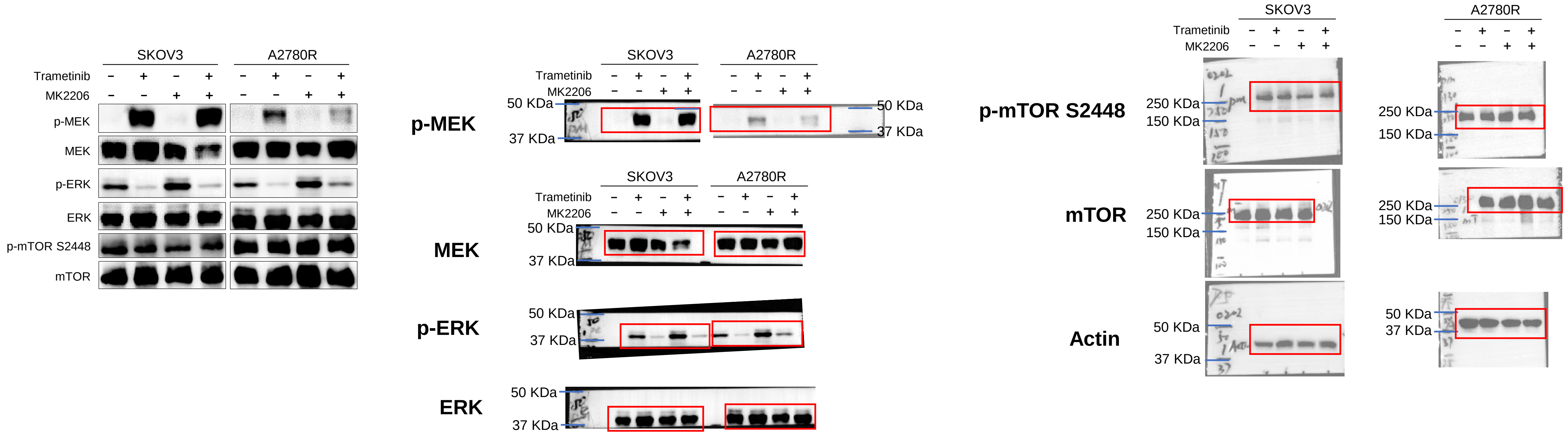

Full unedited gel for **Supplementary Figure 6B**

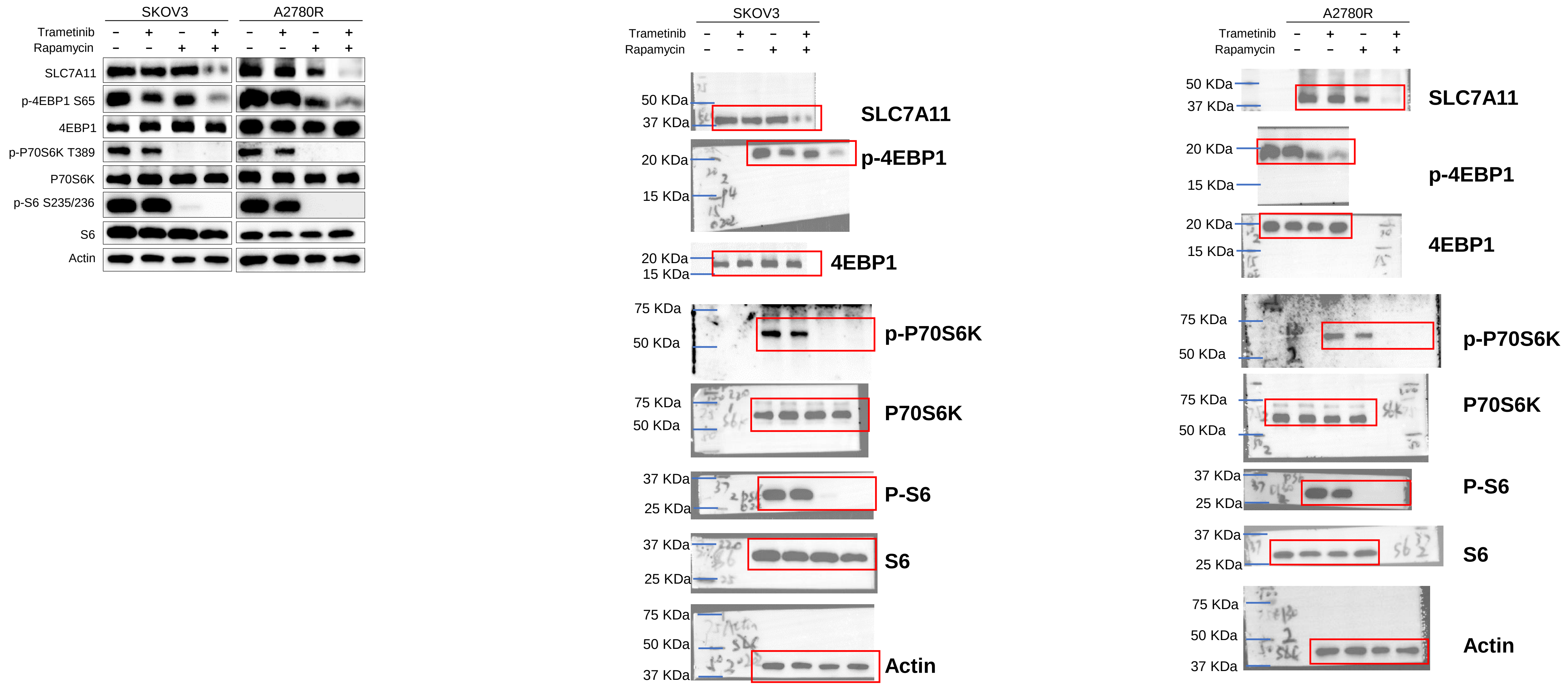

Full unedited gel for **Supplementary Figure 6C**

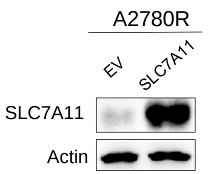

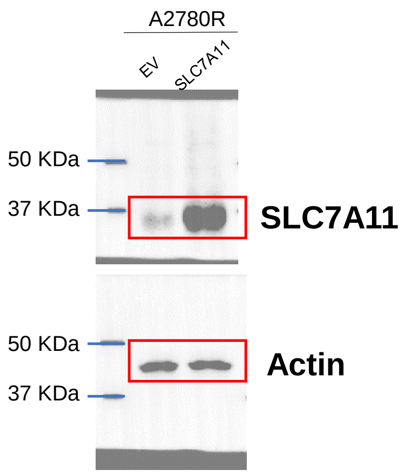

Supplement: Unedited blot and gel images [file jciinsight-9-177857-s048.pdf]
